# Herpes simplex virus type 1 impairs mucosal-associated invariant T cells

**DOI:** 10.1128/mbio.03887-24

**Published:** 2025-03-26

**Authors:** Lauren Stern, Zoe Emanuel, Renee Traves, Katherine Willis, Shivam K. Purohit, Carolyn Samer, Jeffrey Y. W. Mak, David P. Fairlie, David C. Tscharke, Alexandra J. Corbett, Allison Abendroth, Barry Slobedman

**Affiliations:** 1 Infection, Immunity, and Inflammation, School of Medical Sciences, Faculty of Medicine and Health, Sydney, New South Wales, Australia; 2 Charles Perkins Centre, The University of Sydney, Sydney, New South Wales, Australia; 3 ARC Centre of Excellence for Innovations in Peptide and Protein Science, Institute for Molecular Bioscience, University of Queensland, Brisbane, Queensland, Australia; 4 John Curtin School of Medical Research, Australian National University, Canberra, Australian Capital Territory, Australia; 5 Department of Microbiology and Immunology, University of Melbourne at the Peter Doherty Institute for Infection and Immunity, Melbourne, Victoria, Australia; Princeton University, Princeton, New Jersey, USA

**Keywords:** MAIT cell, mucosal-associated invariant T cell, HSV, HSV-1, herpes simplex virus, herpesvirus, immune modulation

## Abstract

Herpes simplex virus type 1 (HSV-1) is a highly successful pathogen that infects mucosal sites and adopts an arsenal of strategies to manipulate host immunity. Mucosal-associated invariant T (MAIT) cells are abundant innate-like T lymphocytes that recognize bacterial and fungal-derived vitamin B-related metabolites presented by major histocompatibility complex class I-related protein 1 (MR1). MAIT cells can also be activated in an MR1-independent manner via cytokine stimulation, predominantly by IL-12 and IL-18. MAIT cell alterations have been identified as being associated with a number of viral infections, but direct interactions between viruses and MAIT cells are poorly understood. It is unknown whether HSV-1 can infect MAIT cells and modulate their functions. Here, we show that HSV-1 can infect primary human MAIT cells, including CD4^±^/CD8^±^ and CD56^±^ MAIT cell subpopulations. Furthermore, HSV-1 infection profoundly inhibits the functional capacity of MAIT cells to respond to T cell receptor (TCR)-dependent stimulation by the MAIT cell activating ligand 5-(2-oxopropylideneamino)-6-D-ribitylaminouracil (5-OP-RU) and to cytokine stimulation by IL-12/IL-18. HSV-1-infected MAIT cells display reduced cytotoxic potential, diminished synthesis of effector cytokines, and decreased expression of key cytokine receptors including IL-18R. In addition, MAIT cells exposed to HSV-1-infected fibroblasts but which remained uninfected (viral GFP-negative) also exhibit a suppressed effector response to TCR-dependent stimulation. The functional suppression of HSV-1-exposed MAIT cells was not mediated by a soluble factor within the supernatant, suggesting direct contact of MAIT cells with HSV-1-infected fibroblasts is required. Overall, this study reveals that HSV-1 can infect MAIT cells and substantially impair MAIT cell effector functions.

**IMPORTANCE:**

Mucosal-associated invariant T cells (MAIT cells) are “unconventional” immune cells that are becoming increasingly appreciated to play important roles in a variety of viral infections. Herpes simplex virus (HSV) causes significant human disease and is a master manipulator of multiple immune functions, but how this virus may control MAIT cells is poorly understood. We discovered that HSV can infect human MAIT cells and impair their functional capacity and also show that MAIT cells exposed to HSV, but which do not show evidence of infection, are similarly impaired. This study therefore identifies an additional immunomodulatory function of HSV.

## INTRODUCTION

Herpes simplex virus type 1 (HSV-1) is a highly prevalent human alphaherpesvirus that infects and replicates at mucosal surfaces (1). HSV-1 infection is often asymptomatic but can cause a range of clinical manifestations, including primary and recurrent vesicular lesions at the orolabial mucosa, genital lesions, ocular infections, and viral encephalitis (2–4). The virus establishes latency in sensory neurons, with reactivation leading to anterograde transport of virions and subsequent productive viral replication at mucocutaneous sites (5, 6).

The response to HSV-1 infection involves both innate and adaptive immunity, with T cells playing a crucial role in controlling viral replication and spread (7). HSV-1 has evolved a variety of immunomodulatory strategies, including those targeted at evasion of T cell immunity, which facilitate viral persistence and lifelong infection of the host (8, 9). HSV-1 encodes functions to inhibit cell-surface major histocompatibility complex (MHC) class I and MHC class II antigen presentation in infected cells, allowing evasion of conventional CD8^+^ T cell and CD4^+^ T cell recognition, respectively (10–13). In addition, HSV-1 has been shown to directly infect both CD4^+^ and CD8^+^ T cells (14) and interfere with T cell receptor (TCR) signaling (15), resulting in reduced cytotoxic capacity and the inhibition of inflammatory cytokine production (16, 17). The targeting of T lymphocyte populations by HSV-1 for functional inhibition also extends to the CD1d-restricted innate-like T cell population, invariant natural killer T (iNKT) cells, whereby HSV-1 suppresses iNKT cell activation and interferon (IFN)-γ secretion in the absence of productive infection (18).

Mucosal-associated invariant T (MAIT) cells are an unconventional innate-like T cell population that is largely uncharacterized in HSV-1 infection. MAIT cells represent 1%–10% of circulating T cells in humans and are also enriched in the skin, liver, and other mucosal tissues, including the oral and respiratory mucosa (19–23). MAIT cells are restricted to the non-classical antigen-presenting molecule, MHC class Ib-related protein 1 (MR1), and express a semi-invariant αβ TCR, which can recognize bacterial and fungal-derived riboflavin metabolites presented on MR1 (24, 25). MAIT cells play a role in antimicrobial responses to riboflavin-synthesizing bacteria and can also contribute to antiviral responses and tissue repair (26–32). While viruses do not encode known MR1 ligands, HSV-1 targets MR1 for degradation and downregulates its surface expression on infected cells, limiting MR1-restricted MAIT TCR activation (33, 34). Although the role of MAIT cells in HSV-1 infection is currently unknown, the HSV-encoded mechanisms for evading immune regulation by MAIT cells suggest that further investigation into direct interactions between HSV-1 and MAIT cells is warranted.

MAIT cells exhibit innate-like immune responsiveness and can be activated through both TCR-dependent and -independent signals (35, 36). TCR-dependent MAIT cell activation occurs via MR1-restricted recognition of microbial-derived non-peptide riboflavin metabolites, such as the potent MAIT cell ligand 5-(2-oxopropylideneamino)-6-D-ribitylaminouracil (5-OP-RU) (37). This elicits a rapid polyfunctional effector response, including the release of pro-inflammatory cytokines such as IFN-γ, tumor necrosis factor (TNF), and interleukin-17 (IL-17), upregulation of lytic granules for target cell killing, and a tissue repair signature (35, 38, 39). MAIT cells can also be activated in a TCR-independent manner through innate cytokines, such as IL-18 in combination with IL-12, IL-15, or type I IFN (36, 40). Cytokine-mediated activation of MAIT cells produces a more limited functional response with heightened expression of perforin, granzyme B, and IFN-γ, corresponding to a Tc1-like phenotype (38). The ability of MAIT cells to be activated by cytokines allows MAIT cells to respond to a variety of infections, including viral infections (41), where cytokines such as IL-18 and IL-12 may be released from activated antigen-presenting cells or virally infected cells. TCR and cytokine signaling in combination promote potent MAIT cell activation and effector function (42, 43).

There is emerging evidence of the relevance of MAIT cells across a range of viral infections, such as influenza virus, severe acute respiratory syndrome coronavirus 2 (SARS-CoV-2), dengue virus, human immunodeficiency virus 1 (HIV-1), and hepatitis viruses (30, 41, 44, 45), including infection of MAIT cells by measles virus (46) and varicella zoster virus (VZV) (47). VZV, an alphaherpesvirus related to HSV-1, was shown to infect MAIT cells *in vitro* and impair multiple MAIT cell functional responses to TCR-dependent and -independent stimulation (48). Whether HSV-1 has the ability to infect human MAIT cells or modulate MAIT cell function has not yet been explored. This study evaluated HSV-1 infection of peripheral blood MAIT cells and identified that MAIT cells are susceptible to infection by HSV-1. We further demonstrate that HSV-1 infection disarms the ability of MAIT cells to respond to TCR-dependent and -independent stimuli *in vitro*, resulting in a profound reduction in cytokine production, cytotoxic potential, and cytokine receptor expression. In addition, MAIT cells exposed to HSV-1-infected cells but which remained uninfected (viral GFP-negative) also display an impaired functional response to TCR-dependent stimulation. These findings define the interaction between HSV-1 and MAIT cells, revealing that these cells are a target of direct infection and functional manipulation by HSV-1.

## RESULTS

### HSV-1 can infect MAIT cells

**Fig 1:**
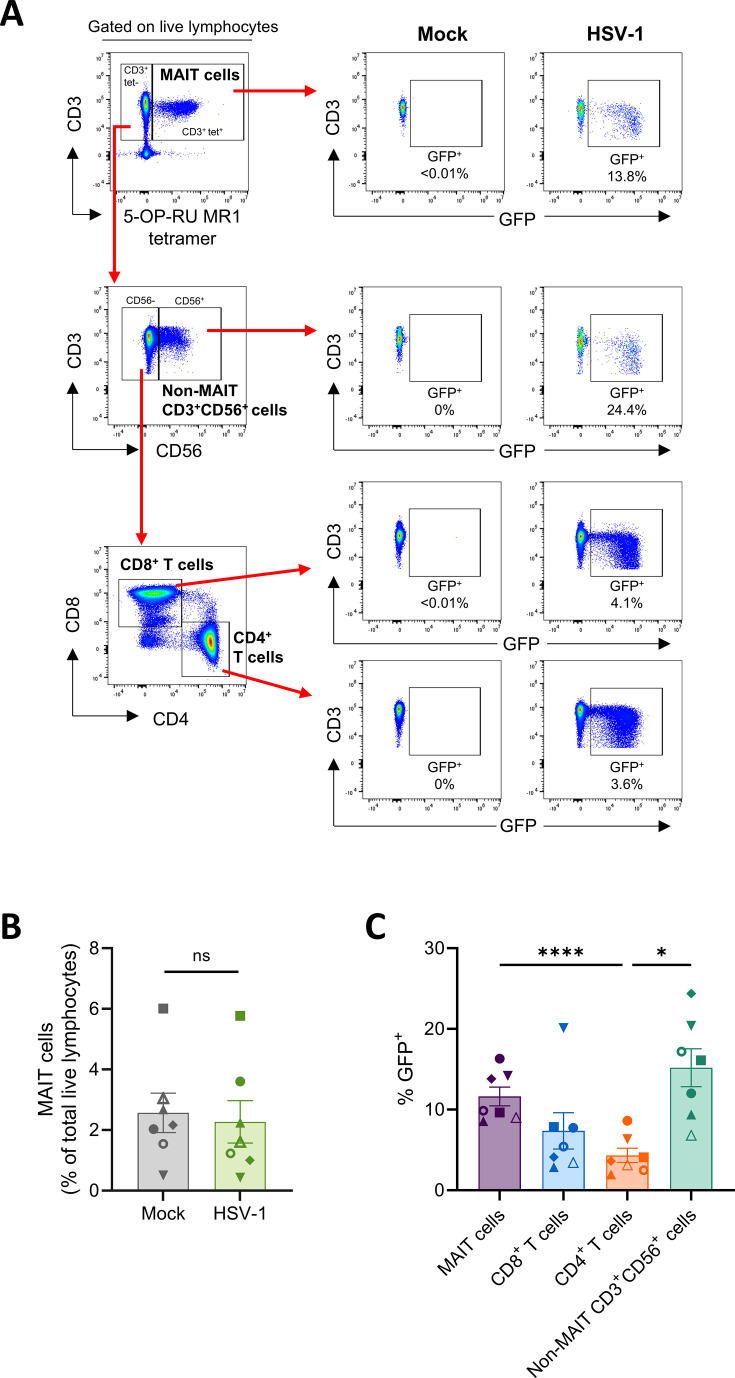
HSV-1 infects human peripheral blood MAIT cells. HFF-hTERTs were mock-infected or infected with an HSV-1 strain expressing GFP under the ICP47 promoter (HSV-1 pICP47_GFP) at a multiplicity of infection (MOI) of 10 for 5 hours. Human PBMCs were co-cultured with mock-infected or HSV-1-infected fibroblasts for 16 hours (ratio of 1 HFF-hTERT:3–5 PBMCs), prior to flow cytometry analysis, with GFP expression used as a marker of infection. (**A**) Representative flow cytometry gating plots identifying MAIT cells (5-OP-RU-MR1 tetramer^+^CD3^+^ lymphocytes), non-MAIT CD3^+^CD56^+^ cells (MR1 tetramer^–^CD3^+^CD56^+^ lymphocytes), CD8^+^ T cells (MR1 tetramer^–^CD3^+^CD56^–^CD4^–^CD8^+^ lymphocytes) and CD4^+^ T cells (MR1 tetramer^–^CD3^+^CD56^–^CD4^+^CD8^–^ lymphocytes) and detection of GFP within these populations. (**B**) Frequencies of MAIT cells (as a percentage of total live lymphocytes) identified by flow cytometry following 16 hours of PBMC co-culture with mock-infected (gray) or HSV-1 pICP47_GFP-infected (green) fibroblasts. Symbols represent individual donors (*n* = 7), mean ± SEM shown. Two-tailed paired *t*-test (ns; non-significant, *P* > 0.05). (**C**) Percentage of MAIT cells (purple), CD8^+^ T cells (blue), CD4^+^ T cells (orange), and non-MAIT CD3^+^CD56^+^ cells (green) expressing GFP following co-culture with HSV-1 pICP47_GFP-infected fibroblasts. Symbols represent individual donors (*n* = 7), and bars represent mean ± SEM. Statistical significance determined by repeated measures one-way analysis of variance (ANOVA) with Tukey’s multiple comparisons test. *P < 0.05 and ****P < 0.0001.

To investigate if HSV-1 could infect primary human MAIT cells, human peripheral blood mononuclear cells (PBMCs) were co-cultured for 16 hours with human telomerase reverse transcriptase immortalized human foreskin fibroblasts (HFF-hTERT) infected with HSV-1. Co-culture of immune cells and HSV-1-infected fibroblasts has been employed in previous studies examining HSV-1 infection of T cells, with efficient viral spread to T cells occurring through the virological synapse (14). We previously utilized a co-culture approach to identify HSV-1 infection of human natural killer (NK) cells by incubating PBMCs with an HSV-1-infected cellular inoculum (49). In this study, we first used an HSV-1 virus that expressed a green fluorescent protein (GFP)/Cre reporter under the promoter for a viral immediate early (IE) gene, infected cell protein (ICP)47 (50), allowing for assessment of infection based on detection of GFP expression by flow cytometry (Fig. 1A).

MAIT cells were identified using flow cytometry by co-staining of CD3 and 5-OP-RU-loaded MR1 tetramer (Fig. 1A; Fig. S1A), which is the gold standard for specific identification of MAIT cells (19, 37). Peripheral blood MAIT cell frequencies ranged from 0.8% to 7.1% of total live CD3^+^ lymphocytes (median 3.5%) in seven healthy adult donors (Fig. S1B), consistent with frequencies reported in previous literature (19, 21). There was no difference in the frequency of live MAIT cells observed between samples incubated with mock-infected or HSV-1-infected fibroblasts (Fig. 1B).

Flow cytometry analysis of viral GFP expression revealed that HSV-1 was able to infect MAIT cells, with GFP detected in a mean of 11.6% (range 8.6%–16.3%) MAIT cells (Fig. 1A and C). Analysis of other CD3^+^ lymphocyte populations showed that a significantly higher percentage of MAIT cells and non-MAIT CD3^+^CD56^+^ lymphocytes (mean 15.2% GFP^+^, range 6.8%–24.4%) were infected compared to non-MAIT CD4^+^ T cells (mean 4.3% GFP^+^, range 2.0%–8.6%) from the same samples (*P* < 0.0001 and *P* = 0.0160, respectively; Fig. 1A and C). HSV-1 infection (GFP^+^) was also detected in a mean of 7.4% of non-MAIT CD8^+^ T cells (range 2.9%–20.1%; Fig. 1A and C). Together, these results confirm that HSV-1 can infect CD4^+^ and CD8^+^ T cells and establish that HSV-1 is capable of infecting primary human peripheral blood MAIT cells, as well as non-MAIT CD3^+^CD56^+^ lymphocytes.

**Fig 2:**
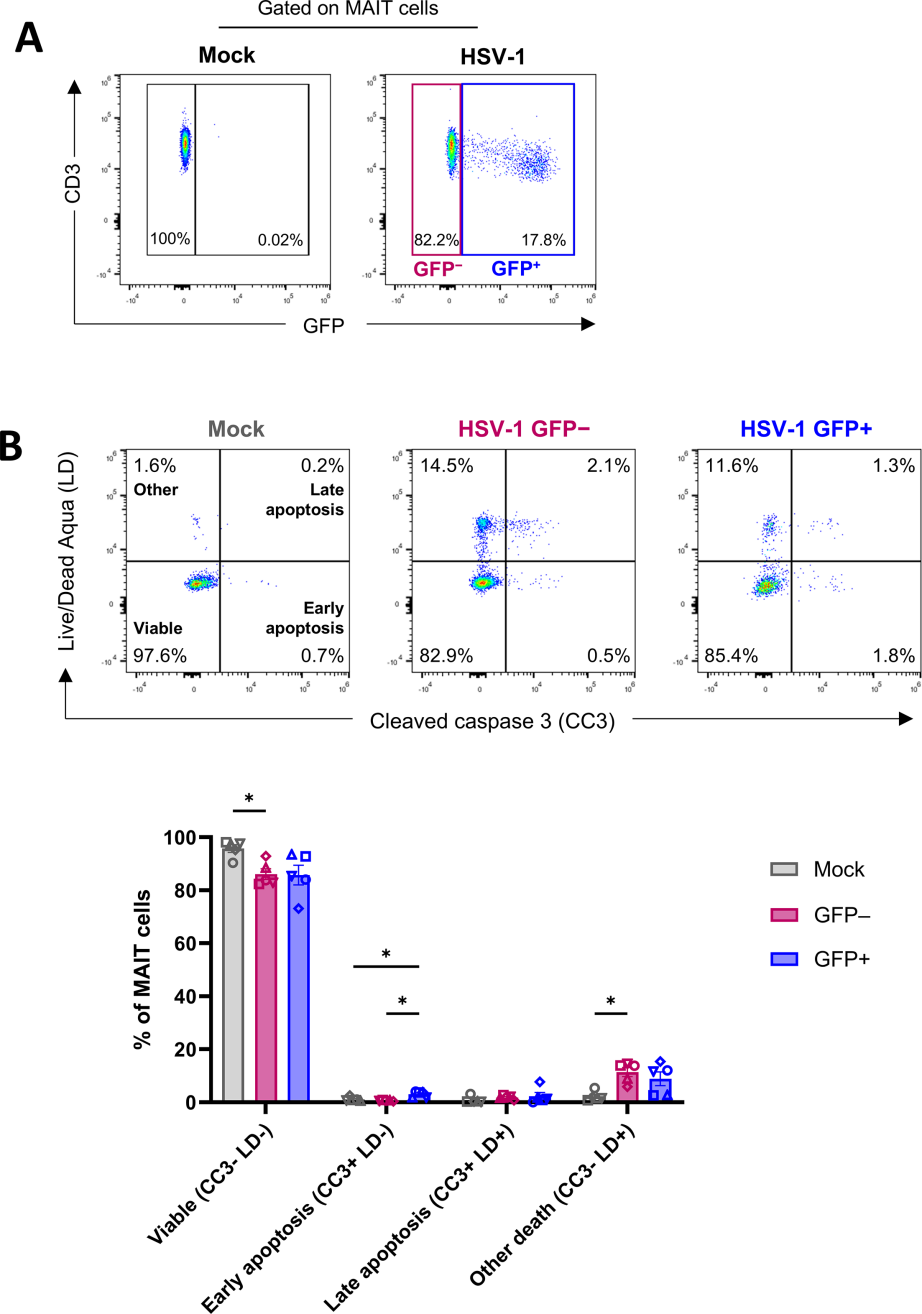
MAIT cell viability following HSV-1 infection. Human PBMCs were co-cultured with mock-infected or HSV-1 pICP47_GFP-infected HFF-hTERTs for 16 hours. PBMCs were then collected and stained with LIVE/DEAD Aqua (LD) viability dye and intracellular anti-cleaved caspase 3 (CC3) antibody to examine MAIT cell viability and apoptosis by flow cytometry. (**A**) MAIT cells (CD3^+^ TCR Vα7.2^+^ CD161^high^ lymphocytes) from HSV-1-infected co-cultures were divided into HSV-1 GFP^+^ and GFP^–^ subpopulations by flow cytometry gating for analysis. Representative gates shown for one donor, displaying the percentage of MAIT cells that were GFP^+^ or GFP^–^. (**B**) MAIT cells were partitioned on the basis of LD and CC3 staining into viable cells (LD^–^ CC3^–^), early apoptotic cells (LD^–^ CC3^+^), late apoptotic cells (LD^+^ CC3^+^), and non-apoptotic non-viable cells (“other” death; LD^+^ CC3^–^). Representative flow cytometry plots (above) and graph (below) depict the percentage of mock-infected (gray), HSV-1 GFP^–^ (pink), and HSV-1 GFP^+^ (blue) MAIT cells in each quadrant. Symbols represent individual PBMC donors (*n* = 5). Bars show mean ± SEM. Statistical significance was determined by repeated measures two-way ANOVA with Tukey’s multiple comparisons test, *P < 0.05.

HSV-1 infection is known to induce apoptosis in some cell types, including T cells (51, 52). To assess the impact of HSV-1 infection on MAIT cell viability, expression of the intracellular apoptotic effector protein, cleaved caspase-3 (CC3), together with amine-reactive LIVE/DEAD viability dye (LD) staining, was measured in MAIT cells using flow cytometry after 16 hours of infection. This analysis enabled the discrimination of viable (CC3^–^/LD^–^), early apoptotic (CC3^+^/LD^–^), late apoptotic (CC3^+^/LD^+^), and non-viable non-apoptotic (other death; CC3^–^/LD^+^) cells (53). MAIT cells from HSV-1-infected co-cultures were divided by flow cytometry gating into GFP^+^ (HSV-1-infected) or GFP^–^ (HSV-1-exposed) subsets (Fig. 2A). While a majority of HSV-1 GFP^–^ (mean 86.0%, range 82.4%–92.9%) and GFP^+^ (85.8%, range 73.1%–93.5%) MAIT cells were viable (CC3^–^/LD^–^), there was a small decline in the percentage of viable cells in both GFP^–^ and GFP^+^ MAIT cell populations, compared to mock-infected MAIT cells (95.8%, range 90.4%–98.1%; *P* = 0.0380 for GFP^–^ and *P* = 0.0835 for GFP^+^, compared to mock; Fig. 2B). A corresponding increase in the proportion of non-viable non-apoptotic (CC3^–^/LD^+^) GFP^–^ MAIT cells (11.5%, range 5.9%–14.5%; *P* = 0.0133) and GFP^+^ MAIT cells (8.9%, range 2.6%–15.4%; *P* = 0.0980) was observed compared to mock-infected MAIT cells (2.3%, range 0.8%–5.4%). There was a low frequency of early apoptotic and late apoptotic cells across all samples (Fig. 2B), with a mean of 3.0% (range 1.8%–4.0%) of GFP^+^ MAIT cells being early apoptotic (CC3^+^/LD^–^), which was significantly higher than the percentage of early apoptotic cells detected in mock-infected (1.2%, range 0.6%–2.5%; *P* = 0.0209) and GFP^–^ (0.6%, range 0.4%–0.9%; *P* = 0.0219) MAIT cells. Overall, MAIT cell viability is largely maintained following co-culture with HSV-1-infected fibroblasts, with a small increase in the frequency of non-viable non-apoptotic MAIT cells at 16 hours post-infection (hpi).

In addition to infecting MAIT cells by co-incubating whole PBMC samples with HSV-1-infected fibroblasts, we investigated whether HSV-1 could infect purified MAIT cells. Primary MAIT cells were first purified from whole PBMC samples by fluorescence-activated cell sorting (FACS), and the isolated MAIT cells were then cultured with HSV-1 pICP47_GFP-infected fibroblasts for 16 hours, at a ratio of 1 fibroblast:3–5 MAIT cells. These experiments led to HSV-1 GFP detection in a mean of 74.8% (range 66.8%–82.4%) MAIT cells (Fig. S2), a substantially higher percentage of GFP^+^ MAIT cells than seen in infection experiments using whole PBMCs (Fig. 1C), where the ratio of MAIT cells to fibroblasts was lower (1 fibroblast:3–5 PBMCs). Thus, HSV-1 can infect a substantial proportion of purified MAIT cells and is capable of infecting MAIT cells in both the presence and absence of other PBMC cell types.

### HSV-1 infects phenotypically distinct MAIT cell subsets

**Fig 3:**
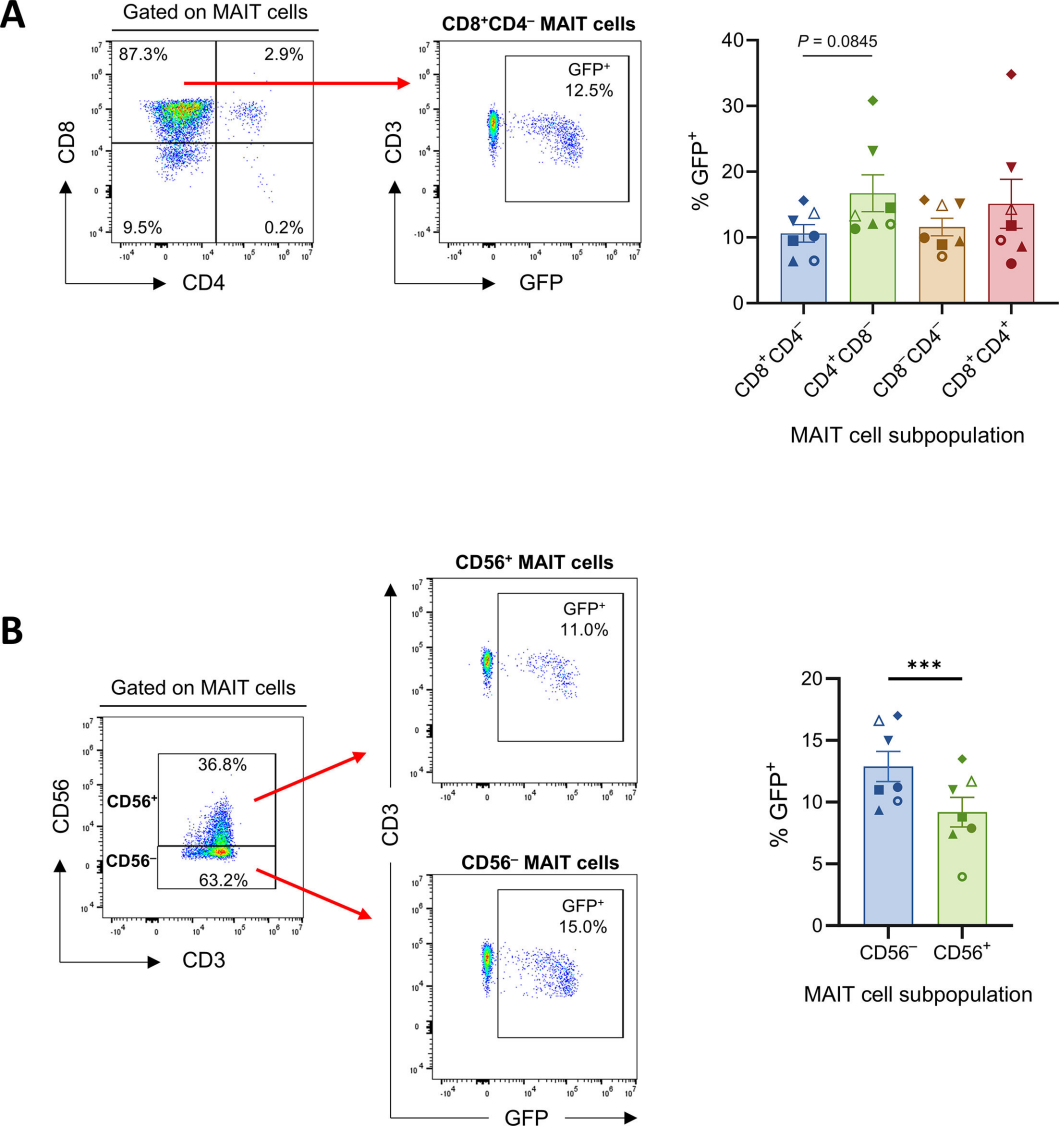
HSV-1 infects multiple peripheral blood MAIT cell subsets. HFF-hTERTs were mock-infected or infected with HSV-1 pICP47_GFP (multiplicity of infection [MOI] of 10) for 5 hours. Human PBMCs were co-cultured with mock- or HSV-1-infected fibroblasts for 16 hours prior to flow cytometry analysis of viral GFP expression in MAIT cell subpopulations. (**A**) MAIT cells (5-OP-RU-MR1 tetramer^+^CD3^+^ lymphocytes) were partitioned into CD8^+^CD4^–^ (blue), CD4^+^CD8^–^ (green), CD8^–^CD4^–^ (brown), and CD8^+^CD4^+^ (red) subsets by flow cytometry gating. Representative flow cytometry plots and graph depict the percentage of GFP expression within each MAIT cell subset. Symbols represent individual donors (*n* = 7), and bars display mean ± SEM. Statistical significance determined by repeated measures one-way ANOVA with Tukey’s multiple comparisons test (all non-significant, *P* > 0.05). (**B**) MAIT cells were divided into CD56^–^ (blue) and CD56^+^ (green) subsets by flow cytometry gating. Representative flow cytometry plots and graph show the percentage of GFP expression within CD56^–^ MAIT cells and CD56^+^ MAIT cells. Symbols represent individual donors (*n* = 7), bars show mean ± SEM. Statistical significance determined by two-tailed paired *t*-test, ***P < 0.001.

MAIT cells can be divided into functionally distinct subpopulations with different phenotypes (54, 55). In human peripheral blood a majority of MAIT cells express CD8, a lower proportion are double negative (CD8^−^CD4^−^), and a minor subset expresses CD4 (54) (Fig. S3A). To investigate if HSV-1 could infect distinct MAIT cell subpopulations, human PBMCs were co-cultured with HSV-1 pICP47_GFP-infected human fibroblasts for 16 hours, after which MAIT cells were divided into CD8^+^CD4^–^, CD8^–^CD4^–^, CD4^+^CD8^–^, and CD8^+^CD4^+^ subsets by flow cytometry gating, and the percentage of viral GFP expression within each subset was measured (Fig. 3A). Whole PBMCs were used for these and subsequent infection experiments as they represent a more biologically relevant condition compared to the use of purified MAIT cells.

The CD8/CD4 subset distribution within the MAIT cell compartment did not differ between samples co-cultured with mock- or HSV-1-infected fibroblasts (Fig. S3A). Analysis of viral GFP in each MAIT cell subpopulation showed that HSV-1 could infect CD8^+^CD4^–^ MAIT cells (mean 10.6% GFP^+^, range 6.4%–15.4%), CD4^+^CD8^–^ MAIT cells (mean 16.7% GFP^+^, range 11.3%–30.8%), CD8^–^CD4^–^ MAIT cells (mean 11.7% GFP^+^, range 7.1%–15.7%), and CD8^+^CD4^+^ MAIT cells (mean 15.1% GFP^+^, range 6.0%–34.8%; Fig. 3A), with a trend toward a higher percentage of infection in CD4^+^CD8^–^ compared to CD8^+^CD4^–^ MAIT cells (*P* = 0.0845; Fig. 3A). CD56 cell-surface expression on MAIT cells is associated with enhanced responses to activatory stimuli, including innate cytokines (56, 57). To assess whether HSV-1 could infect both CD56^−^ and CD56^+^ MAIT cell subpopulations, total MAIT cells were partitioned into CD56^−^ and CD56^+^ subsets by flow cytometry gating, and viral GFP expression in each subset was examined (Fig. 3B). HSV-1 was able to infect both CD56^−^ and CD56^+^ MAIT cells, with a greater proportion of CD56^−^ MAIT cells infected (mean 12.9% GFP^+^, range 9.4%–17.0%) compared to CD56^+^ MAIT cells (mean 9.2% GFP^+^, range 4.0%–13.5%; *P* = 0.0006; Fig. 3B). There was no difference in the frequency of CD56^+^ MAIT cells between mock- and HSV-1-infected samples (Fig. S3B), and the viability of CD56^−^ and CD56^+^ MAIT cells from HSV-1 co-cultures was similar (Fig. S4), indicating that exposure to HSV-1 did not differentially impact the survival of CD56^−^ or CD56^+^ MAIT cells. Together, these results indicate that HSV-1 is capable of infecting multiple MAIT cell subsets.

### HSV-1-infected MAIT cells express immediate-early, early, and late viral genes

**Fig 4:**
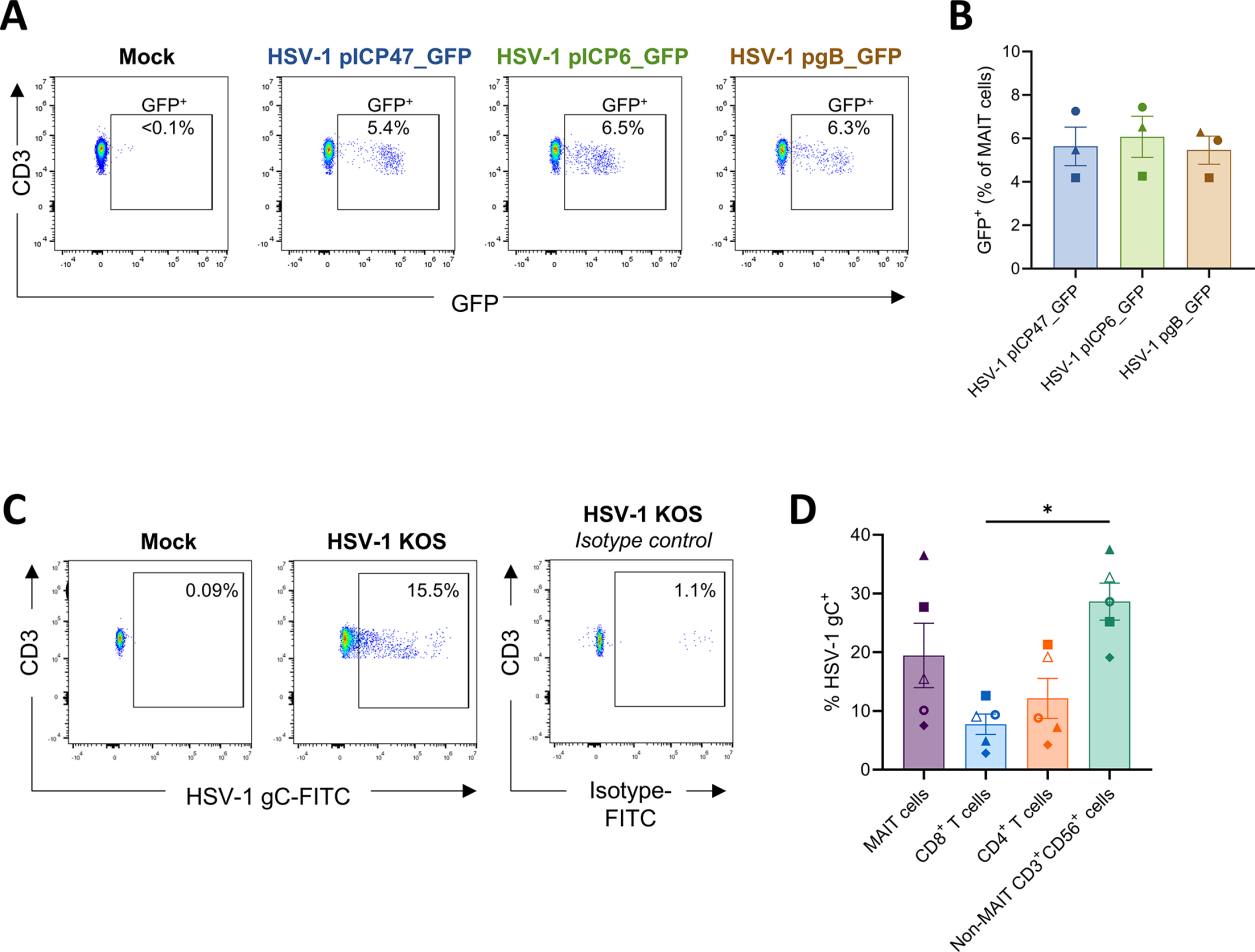
HSV-1 expresses immediate early, early, and late viral genes in human peripheral blood MAIT cells. (**A and B**) HFF-hTERTs were mock-infected or infected for 20 hours (MOI of 5) with HSV-1 strains expressing GFP under the promoter for viral gene ICP47 (immediate early gene), ICP6 (early gene), or gB (leaky late gene), respectively. Human PBMCs were co-cultured with mock-infected or HSV-1-infected fibroblasts for 1 day, prior to harvesting for flow cytometry to assess the percentage of GFP^+^ MAIT cells. (**A**) Representative flow cytometry plots and (**B**) graph show the percentage of GFP in MAIT cells (5-OP-RU-MR1 tetramer^+^CD3^+^ lymphocytes) with each virus strain; HSV-1 pICP47_GFP (blue), HSV-1 pICP6_GFP (green), and HSV-1 pgB_GFP (brown). Symbols represent individual PBMC donors (*n* = 3). Bars show mean ± SEM. Repeated measures one-way ANOVA with Tukey’s multiple comparisons test (all comparisons non-significant, *P* > 0.05). (**C and D**) HFF-hTERTs were mock-infected or infected with HSV-1 strain KOS (MOI of 10) for 5 hours. PBMCs were then co-cultured with mock- or HSV-1-infected HFF-hTERTs for 16 hours, before being collected and stained with an anti-HSV-1 glycoprotein C (gC) antibody conjugated to fluorescein isothiocyanate (FITC) for flow cytometry analysis. (**C**) Representative flow cytometry plots show the percentage of HSV-1 gC^+^ MAIT cells in PBMC samples co-cultured with mock-infected or HSV-1 KOS-infected fibroblasts. FITC-conjugated isotype control antibody staining in MAIT cells co-cultured with HSV-1 KOS-infected fibroblasts co-culture is shown on the right. (**D**) The percentage of MAIT cells (5-OP-RU-MR1 tetramer^+^CD3^+^ lymphocytes; purple), CD8^+^ T cells (MR1 tetramer^–^CD3^+^CD56^–^CD4^–^CD8^+^ lymphocytes; blue), CD4^+^ T cells (MR1 tetramer^–^CD3^+^CD56^–^CD4^+^CD8^–^ lymphocytes; orange), and non-MAIT CD3^+^CD56^+^ cells (MR1 tetramer^–^CD3^+^CD56^+^ lymphocytes; green) expressing HSV-1 gC GFP after co-culture of PBMCs with HSV-1-infected fibroblasts for 16 hours. Symbols represent individual PBMC donors (*n* = 5). Bars show mean ± SEM. Repeated measures one-way ANOVA with Tukey’s multiple comparisons test, *P < 0.05.

A hallmark of the herpesvirus productive replicative cycle is a temporally regulated cascade of viral gene expression with IE, early, leaky late, and true late kinetic phases (58, 59). Expression of viral IE genes commences within hours post-infection and synthesis of late gene products peaks at approximately 10–16 hpi (60, 61). Having initially identified infection of MAIT cells using an HSV-1 virus that expressed GFP under a viral IE gene promoter (pICP47; Fig. 1A), we next investigated whether MAIT cells could support subsequent phases of HSV-1 gene expression. To assess this, human PBMCs were co-cultured for 1 day with fibroblasts infected with recombinant HSV-1 viruses engineered to express GFP under the promoter for an IE (ICP47), early (ICP6), or a leaky late (glycoprotein B [gB]) viral gene, respectively, at a multiplicity of infection (MOI) of 5. Examination of GFP expression by flow cytometry revealed that a similar percentage of MAIT cells were GFP positive across all three viruses (mean 5.6%, 6.1%, and 5.5% GFP^+^ for IE, early, and leaky late viral promoters, respectively; Fig. 4A and B), indicating that MAIT cells are capable of supporting the expression of distinct temporal viral gene classes during HSV-1 infection.

Incubation of PBMCs in the supernatant collected from HSV-1-infected HFF-hTERT cells or HFF-hTERT:PBMC co-cultures at 16 hpi did not lead to detectable GFP expression in MAIT cells (Fig. S5), a finding not surprising given that any cell-free HSV-1 infects T cells with very low efficiency (14). Further, these data show that the detection of HSV-1 GFP expression in MAIT cells following co-culture with HSV-1-infected fibroblasts (Fig. 1) did not result from the uptake of free GFP within the supernatant. Nuclear localization of the GFP signal in HSV-1 pICP47_GFP (HSV-1 pICP47_eGFP/Cre)-infected MAIT cells was also observed by fluorescence microscopy (Fig. S6), consistent with the expected localization of the eGFP/Cre fusion protein as the Cre recombinase drives nuclear localization (62). These findings together suggest that the detection of HSV-1 promoter-driven GFP expression in MAIT cells was a result of GFP expression in MAIT cells, rather than the external binding or internalization of GFP by MAIT cells.

To extend our observations of MAIT cell infection by HSV-1, expression of the HSV-1 envelope glycoprotein C (gC) on MAIT cells was examined using anti-gC antibody staining and flow cytometry. HSV-1 gC (UL44) is a true late (γ_2_) viral protein whose expression is dependent on viral DNA replication (63). After 16 hours of PBMC co-culture with HSV-1-infected fibroblasts, cell-surface gC was detected on a mean of 19.5% (range 7.5%–36.5%) MAIT cells (Fig. 4C and D), demonstrating that MAIT cells express a true late HSV-1 protein following HSV-1 infection. The percentage of HSV-1 gC^+^ MAIT cells was slightly higher but not significantly different compared to the percentage of GFP^+^ MAIT cells detected using the HSV-1 pICP47_GFP virus (Fig. S7). These results confirm that MAIT cells can support multiple classes of HSV-1 gene expression, including the expression of a true late viral protein. Within the T lymphocyte compartment, HSV-1 gC expression was also detected in a proportion of non-MAIT CD3^+^CD56^+^ lymphocytes (mean 28.6%, range 19.1%–37.5%), non-MAIT CD8^+^ T cells (7.8%, range 2.8%–12.6%) and non-MAIT CD4^+^ T cells (12.1%, range 4.3%–21.3%), with a significantly higher proportion of non-MAIT CD3^+^CD56^+^ lymphocytes expressing gC^+^ compared to non-MAIT CD8^+^ T cells (*P* = 0.0127; Fig. 4D).

### HSV-1-infected MAIT cells can transmit infectious virus to fibroblasts

**Fig 5:**
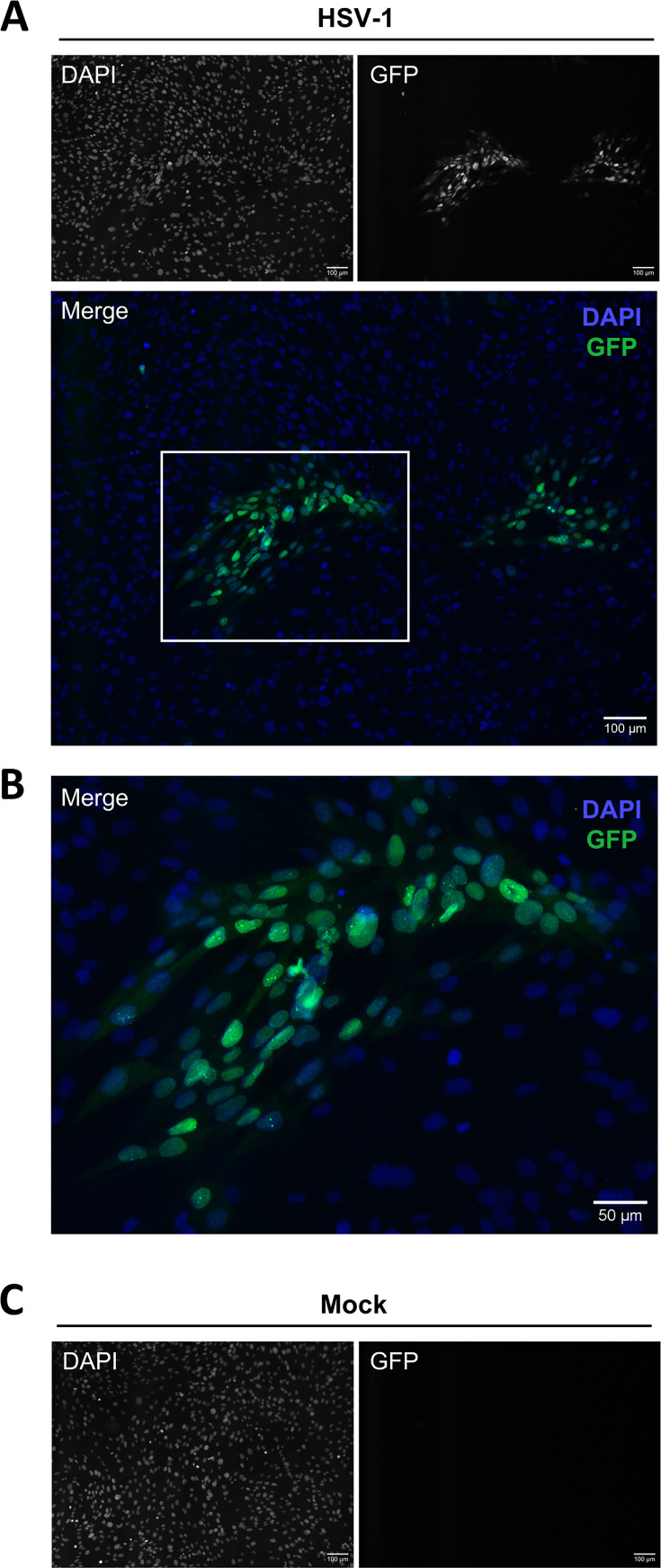
HSV-1-infected MAIT cells can transmit infectious virus to fibroblasts. GFP^+^ MAIT cells (CD3^+^ 5-OP-RU-MR1 tetramer^+^ live lymphocytes) were isolated by FACS from PBMCs after 16 hours of co-culture with HSV-1 pICP47_GFP-infected HFF-hTERTs. Isolated GFP^+^ MAIT cells were treated with low-pH citrate buffer and then added to uninfected HFF-hTERT monolayers at a ratio of 1 MAIT cell to 20 HFF-hTERTs for 1 day. After 1 day, monolayers were washed, fixed, and counterstained with 4′,6-diamidino-2-phenylindole (DAPI), and GFP^+^ infectious centers were detected and visualized by fluorescence microscopy. (**A**) Representative image of GFP^+^ infectious centers detected in the HFF-hTERT monolayer after 1 day of incubation with GFP^+^ MAIT cells isolated from HSV-1-infected co-cultures. Single-channel grayscale images for DAPI and GFP shown above, with the merge image of DAPI (blue) and GFP (green) underneath. The region indicated by the white box is shown enlarged in panel **B**, with DAPI in blue and GFP in green. (**C**) Representative single-channel grayscale images of DAPI and GFP in the mock-infected HFF-hTERT monolayer. Images are representative of three independent donors.

Having identified that HSV-1 could infect peripheral blood MAIT cells, we next sought to determine if HSV-1-infected MAIT cells were capable of transmitting HSV-1 infection to other susceptible cells. An infectious center assay (47, 64, 65) was performed to investigate the ability of HSV-1-infected MAIT cells to transfer HSV-1 infection to a monolayer of fibroblasts. PBMCs were co-cultured with HSV-1 pICP47_GFP-infected HFF-hTERT cells for 16 hours, after which HSV-1 GFP^+^ MAIT cells were isolated by FACS and washed in low-pH citrate buffer to inactivate any unpenetrated surface-bound virions (66–69). Citrate buffer-treated GFP^+^ MAIT cells were then incubated with uninfected HFF-hTERT monolayers for 1 day. After 1 day, multiple distinct GFP^+^ infectious centers were visible in the fibroblast monolayers by fluorescence microscopy (Fig. 5A and B), demonstrating the transmission of infectious virus from MAIT cells to fibroblasts. No evidence of infection was detected in mock-infected monolayers (Fig. 5C). These results show that MAIT cells can support the transmission of infectious HSV-1 virus to fibroblasts.

### HSV-1 suppresses MAIT cell effector cytokine responses to TCR-dependent and cytokine-mediated stimuli

HSV-1 is known to inhibit the effector functions of several lymphocyte subsets *in vitro*, including NK cells (49), iNKT cells (18), and conventional CD8^+^ T cells (16). We next sought to investigate the functional effects of HSV-1 infection on MAIT cells by examining MAIT cell effector responses to TCR dependent and TCR independent, i.e., cytokine- mediated, stimulation following co-culture with HSV-1-infected fibroblasts. Human PBMCs were harvested following 16 hours of co-culture with mock- or HSV-1 pICP47_GFP-infected fibroblasts and were stimulated with either the potent MR1-TCR ligand 5-OP-RU for 6 hours, the cytokines IL-18 and IL-12 together (IL-12/IL-18) for 20 hours, or a combination of IL-12/IL-18 and 5-OP-RU (IL-12/IL-18 + 5-OP-RU) for 20 hours, for synergistic TCR and cytokine-mediated activation. These stimulation conditions reflect the differing activation kinetics of MAIT cells in response to TCR or cytokine signals, as TCR-dependent activation induces a more rapid effector response in MAIT cells compared to cytokine-driven activation (38).

**Fig 6:**
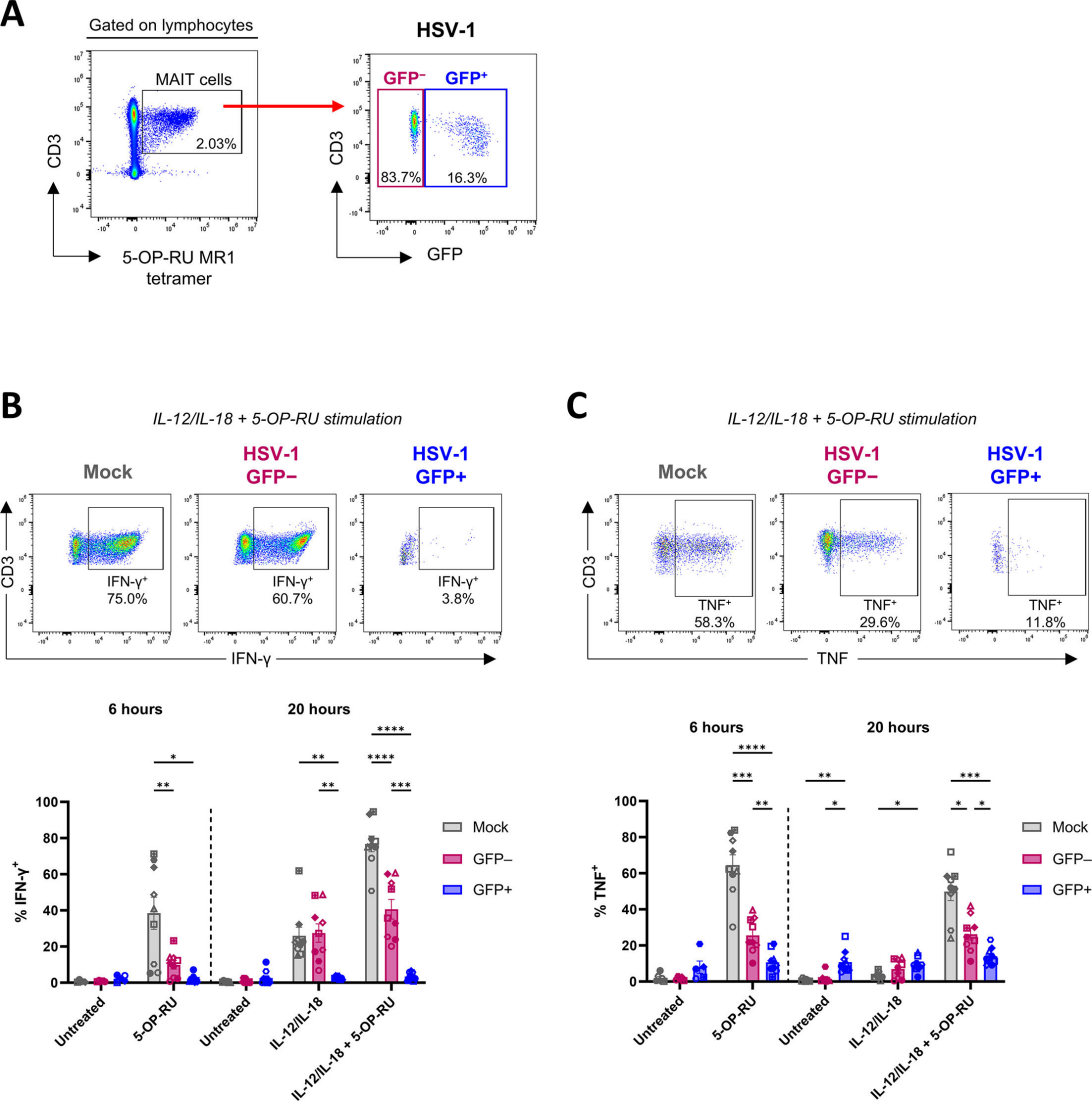
IFN-γ and TNF expression by MAIT cells is impaired by HSV-1. HFF-hTERTs were mock-infected or infected with HSV-1 pICP47_GFP (MOI of 10) for 5 hours. Human PBMCs were co-cultured with mock-infected or HSV-1 pICP47_GFP-infected fibroblasts for 16 hours. PBMCs were then removed from co-culture and treated with either 5-OP-RU (10 nM) for 6 hours, IL-12/IL-18 (both 50 ng/mL) for 20 hours, or IL-12/IL-18 + 5-OP-RU for 20 hours, with corresponding unstimulated controls, prior to intracellular flow cytometry analysis for IFN-γ and TNF expression in MAIT cells. (**A**) MAIT cells (defined as 5-OP-RU-MR1 tetramer^+^CD3^+^ lymphocytes) from PBMC samples co-cultured with HSV-1-infected fibroblasts were divided by flow cytometry gating into GFP^+^ and GFP^–^ subsets for analysis after stimulation. Representative gating strategy shown. (**B and C**) Representative flow cytometry plots of IL-12/IL-18 + 5-OP-RU treatment (above) and graphs (below) display the percentage of mock (gray), HSV-1 GFP^–^ (pink), or HSV-1 GFP^+^ (blue) MAIT cells expressing (**B**) IFN-γ and (**C**) TNF, following the indicated stimulations. Symbols represent individual donors (*n* = 9), and bars represent mean ± SEM. Statistical significance was determined by repeated measures two-way ANOVA with Tukey’s multiple comparisons test, *P < 0.05, **P < 0.01, ***P < 0.001, and ****P < 0.0001.

Using flow cytometry, expression of pro-inflammatory cytokines IFN-γ and TNF by MAIT cells following TCR-dependent and/or cytokine stimulation was analyzed (Fig. 6). After gating on live (viability dye negative [LD^–^]) MAIT cells (Fig. S1A), MAIT cells co-cultured with HSV-1-infected fibroblasts were divided by flow cytometry gating into GFP^+^ (HSV-1-infected) or GFP^–^ (HSV-1-exposed) subsets (Fig. 6A) to enable assessment of any differences in functional responses between these populations, in comparison to mock-infected MAIT cells.

IFN-γ expression was induced in mock-infected MAIT cells following stimulation with the TCR ligand 5-OP-RU, cytokines IL-12/IL-18, or combined treatment (IL-12/IL-18 + 5-OP-RU; Fig. 6B). Strikingly, IFN-γ expression following TCR-mediated activation with 5-OP-RU was profoundly suppressed in both HSV-1 GFP^+^ and GFP^–^ MAIT cells (19.7- and 3.8-fold lower than mock, respectively; *P* = 0.0108 and *P* = 0.0099; Fig. 6B). In response to stimulation with IL-12/IL-18, a similar percentage of IFN-γ expression was induced in mock-infected (mean 25.9%, range 15.2%–61.9%) and HSV-1 GFP^–^ MAIT cells (mean 27.5%, range 6.7%–48.7%; *P* = 0.9503), while HSV-1 GFP^+^ MAIT cells again failed to upregulate IFN-γ (mean 2.7% IFN-γ^+^, range 1.5%–3.9%; *P* = 0.0033 relative to mock, *P* = 0.0030 relative to GFP^–^; Fig. 6B). HSV-1 GFP^+^ MAIT cells also did not respond to combined stimulation with IL-12/IL-18 + 5-OP-RU (mean 3.4% IFN-γ^+^, range 0.8%–6.5%; *P* < 0.0001 relative to mock, *P* = 0.0004 relative to GFP^–^ MAIT cells; Fig. 6B). GFP^–^ MAIT cells displayed a lower percentage of IFN-γ^+^ cells compared to mock-infected MAIT cells in response to combined stimulation (mean 40.6%, range 20.1%–60.7% vs 76.8%, range 50.8%–94.5%; *P* < 0.0001), and combined stimulation induced a relatively small increase in IFN-γ expression in GFP^–^ MAIT cells compared to IL-12/IL-18 stimulation alone (1.5-fold increase in IFN-γ^+^ cells in GFP^–^ vs 3.0-fold increase in IFN-γ^+^ mock-infected MAIT cells, between IL-12/IL-18 alone and combined treatment; Fig. 6B).

Overall, HSV-1 infection of MAIT cells resulted in a profound inhibition of IFN-γ expression in response to TCR-dependent and -independent stimulation. HSV-1 GFP^–^ MAIT cells from the same cultures retained the capacity to respond to IL-12/IL-18 but also showed an impaired ability to upregulate IFN-γ in response to TCR-mediated and combination stimulation.

Analysis of TNF expression revealed a similar pattern following 5-OP-RU or combination (IL-12/IL-18 + 5-OP-RU) stimulation, with robust TNF expression in mock-infected MAIT cells and significantly lower TNF expression in GFP^–^ and GFP^+^ MAIT cells relative to mock (Fig. 6C). The percentage of GFP^+^ MAIT cells expressing TNF was also significantly lower than in GFP^–^ MAIT cells after 5-OP-RU stimulation (2.4-fold lower; *P* = 0.0085) and combination stimulation (1.8-fold lower; *P* = 0.0234; Fig. 6C). Limited TNF upregulation was observed in IL-12/IL-18 stimulated samples from all groups (Fig. 6C), which was expected as TCR-dependent MAIT cell activation is needed for robust TNF production (38, 56). Interestingly, a higher percentage of GFP^+^ MAIT cells expressed TNF in the untreated control at 20 hours compared to mock-infected (*P* = 0.0052) and GFP^–^ (*P* = 0.0131) MAIT cells (13.7- and 6.9-fold higher, respectively), as well as in the IL-12/IL-18 stimulated condition compared to mock-infected MAIT cells (2.8-fold higher; *P* = 0.0159; Fig. 6C).

Overall, these findings demonstrate that TNF upregulation was suppressed in GFP^+^ MAIT cells, and to a lesser degree in GFP^–^ MAIT cells, in response to TCR-dependent and combination stimulation. Thus, HSV-1 impaired the ability of MAIT cells to upregulate multiple pro-inflammatory cytokines in response to activating stimuli, with the greatest impairment evident in GFP^+^ MAIT cells.

### HSV-1 suppresses the cytotoxic potential of MAIT cells

Upon activation, MAIT cells can release cytotoxic granule proteins, including granzyme B and perforin, which mediate the killing of target cells (43). Having observed the potent suppression of cytokines in MAIT cells following co-culture with HSV-1-infected fibroblasts, we examined whether HSV-1 impacts the ability of MAIT cells to degranulate following TCR-dependent or cytokine-mediated stimulation. Using flow cytometry, expression of the degranulation marker CD107a was assessed in mock-infected, GFP^+^, and GFP^–^ MAIT cells following stimulation with 5-OP-RU or IL-12/IL-18 or their combination (IL-12/IL-18 + 5-OP-RU).

**Fig 7:**
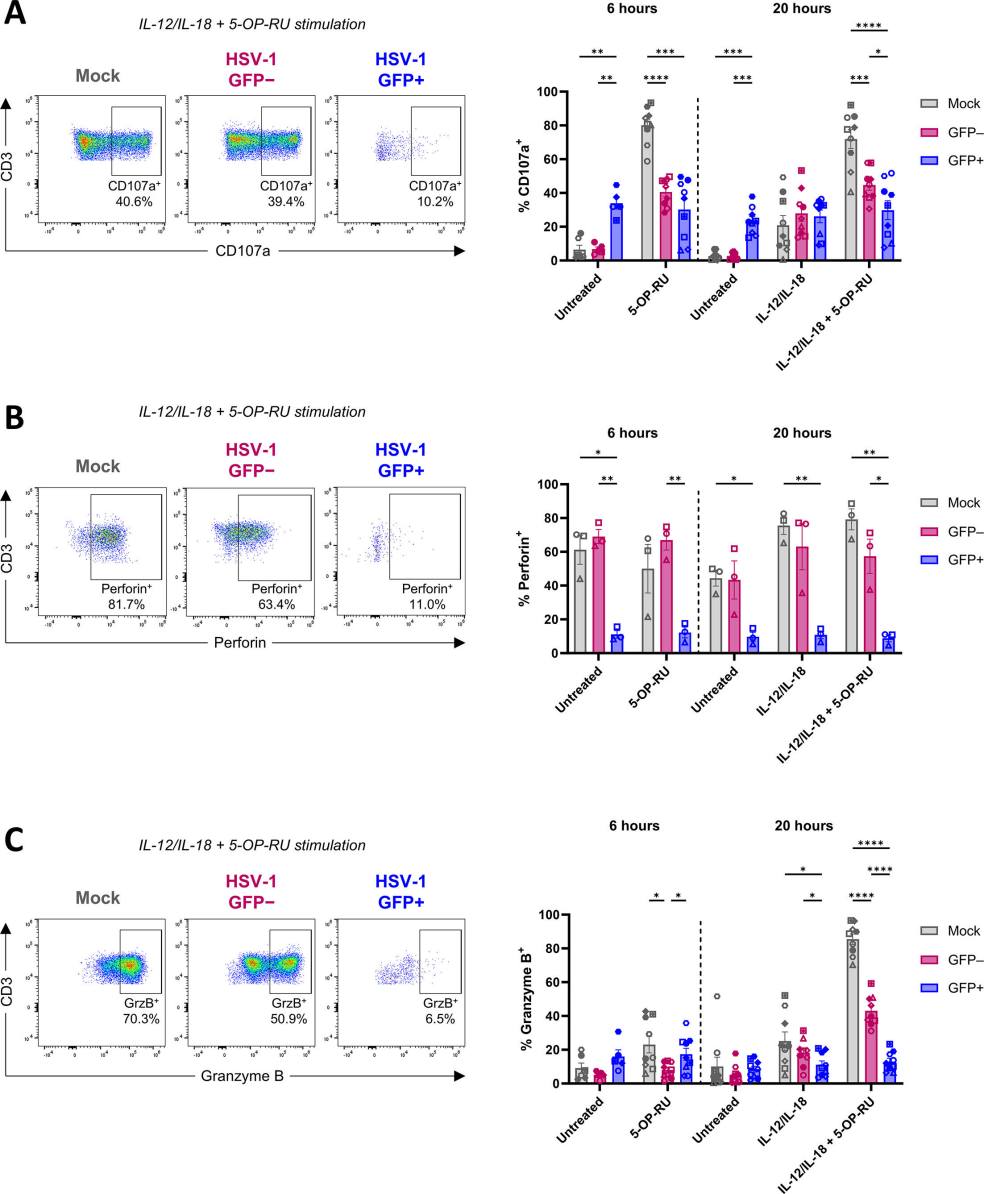
HSV-1 suppresses MAIT cell expression of CD107a, perforin, and granzyme B. HFF-hTERTs were mock-infected or infected with HSV-1 pICP47_GFP (MOI of 10) for 5 hours. Human PBMCs were co-cultured with mock- or HSV-1-infected fibroblasts for 16 hours, and then PBMCs were removed from co-culture and treated with either 5-OP-RU (10 nM) for 6 hours, IL-12/IL-18 (both 50 ng/mL) for 20 hours, or IL-12/IL-18 + 5-OP-RU for 20 hours, prior to flow cytometry analysis. MAIT cells (5-OP-RU-MR1 tetramer^+^CD3^+^ lymphocytes) from HSV-1-infected co-cultures were divided by flow cytometry gating into GFP^+^ and GFP^–^ subsets for analysis. Representative flow cytometry plots of IL-12/IL-18 + 5-OP-RU treatment (left) and graphs (right) display the percentage of mock (gray), HSV-1 GFP^–^ (pink), or HSV-1 GFP^+^ (blue) MAIT cells expressing (**A**) CD107a (*n* = 9), (**B**) perforin (*n* = 3), and (**C**) granzyme B (GrzB; *n* = 9). Symbols represent individual donors, and bars display mean ± SEM. Statistical significance was determined by repeated measures two-way ANOVA with Tukey’s multiple comparisons test comparing mock, GFP^–^, and GFP^+^ MAIT cells within each treatment group, *P < 0.05, **P < 0.01, ***P < 0.001, and ****P < 0.0001.

Interestingly, in the untreated control at both time points, the percentage of GFP^+^ MAIT cells expressing CD107a was significantly higher compared to both mock-infected (*P* = 0.0094 at 6 hours; *P* = 0.0002 at 20 hours) and GFP^–^ MAIT cells (*P* = 0.0025 at 6 hours; *P* = 0.0001 at 20 hours; Fig. 7A), possibly reflecting increased exocytosis or lysosomal trafficking of CD107a in GFP^+^ MAIT cells. The percentage of GFP^+^ MAIT cells expressing CD107a did not increase following stimulation with 5-OP-RU and/or IL-12/IL-18, contrasting with increased degranulation observed in mock-infected and GFP^–^ MAIT cells after stimulation (Fig. 7A). MAIT cell degranulation in response to 5-OP-RU and combination stimulation was significantly lower in both GFP^+^ and GFP^–^ MAIT cells relative to mock-infected MAIT cells, and was also significantly lower in GFP^+^ MAIT cells compared to GFP^–^ MAIT cells following combination stimulation (Fig. 7A).

Next, we assessed granzyme B and perforin expression in mock-infected, GFP^+^, and GFP^–^ MAIT cells using intracellular flow cytometry. The percentage of GFP^+^ MAIT cells expressing perforin was markedly lower compared to both GFP^–^ and mock-infected MAIT cells under all conditions examined (Fig. 7B). Granzyme B expression was strongly induced in mock-infected MAIT cells when stimulated with IL-12/IL-18 + 5-OP-RU (8.5-fold increase relative to untreated; *P* < 0.0001; Fig. 7C). The percentage of GFP^+^ MAIT cells expressing granzyme B was significantly lower relative to both mock-infected and GFP^–^ MAIT cells in response to IL-12/IL-18 and combination stimulation (Fig. 7C). Furthermore, a significantly lower percentage of GFP^–^ MAIT cells expressed granzyme B relative to mock-infected MAIT cells following combination stimulation (*P* < 0.0001), as well as relative to both mock-infected (*P* = 0.0128) and GFP^+^ MAIT cells (*P* = 0.0364) following stimulation with 5-OP-RU (Fig. 7C). Taken together, these results show that HSV-1 suppresses the cytotoxic potential of MAIT cells, evidenced by reduced degranulation and lower cytotoxic granule content relative to mock-infected MAIT cells following stimulation.

### HSV-1 suppression of MAIT cell effector functions is not mediated by a secreted factor in co-culture supernatant

**Fig 8:**
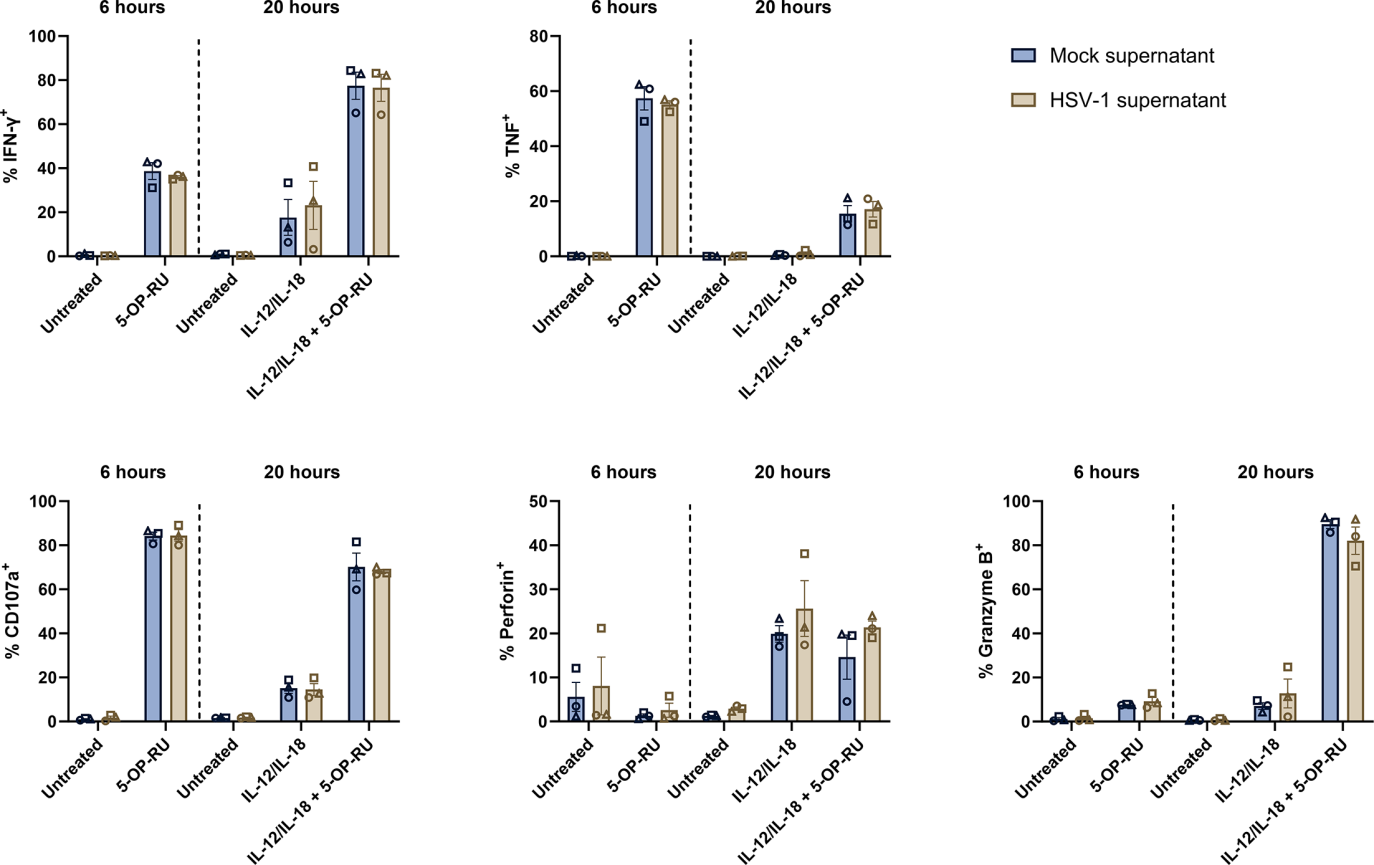
Functional inhibition of MAIT cells by HSV-1 is not mediated by a soluble factor in co-culture supernatant. Fresh autologous human PBMCs were incubated for 24 hours in supernatants derived from the 16-hour co-culture of mock- or HSV-1 pICP47_GFP-infected fibroblasts and PBMCs. PBMCs were then left untreated or stimulated with 5-OP-RU (10 nM, for 6 hours), IL-12/IL-18 (50 ng/mL each, for 20 hours), or IL-12/IL-18 + 5-OP-RU (for 20 hours), prior to flow cytometry analysis. Graphs display the percentage of IFN-γ^+^, TNF^+^, CD107a^+^, perforin^+^, or granzyme B^+^ MAIT cells (5-OP-RU-MR1 tetramer^+^CD3^+^ lymphocytes) after incubation in mock (blue) or HSV-1 (brown) co-culture supernatant and subsequent stimulation. Symbols represent individual PBMC donors (*n* = 3), and bars display mean ± SEM. *P*-values determined by repeated measures two-way ANOVA with Šídák’s multiple comparisons test comparing mock and HSV-1 supernatant (all comparisons were not significant; *P* > 0.05).

Having established that HSV-1 functionally impaired both GFP^+^ and GFP^–^ MAIT cells across a range of outputs, we explored whether a secreted factor was responsible for this inhibition. To test this, supernatant from the 16-hour co-culture of PBMCs and mock- or HSV-1 pICP47_GFP-infected fibroblasts was collected and incubated with fresh autologous PBMCs for 1 day, after which PBMCs were stimulated with 5-OP-RU and/or IL-12/IL-18. As noted previously, no GFP expression was detected in MAIT cells after incubation in supernatant from HSV-1-infected co-cultures (Fig. S5A). Importantly, across all stimulations, flow cytometry analysis revealed no difference in the percentage of MAIT cells expressing IFN-γ, TNF, CD107a, perforin, or granzyme B when incubated with mock or HSV-1 co-culture supernatant (Fig. 8). These data suggest that the functional inhibition seen in GFP^–^ MAIT cells (Fig. 6; Fig. 7) is not mediated by a secreted factor present in the HSV-1 co-culture supernatant, but rather may be dependent on direct contact between the HSV-1-infected fibroblasts and MAIT cells.

### Expression of cytokine receptors and activation markers by MAIT cells is impaired by HSV-1

Our findings that HSV-1 infection inhibited MAIT cell cytokine and cytotoxic responses to multiple stimuli led us to explore whether the expression of key cytokine receptors required for cytokine-mediated MAIT cell activation was impacted by HSV-1. Using flow cytometry, we examined the surface expression of IL-18 receptor α (IL-18Rα) on MAIT cells following 16-hour co-culture with mock- or HSV-1 pICP47_GFP-infected fibroblasts (16 hpi), or after subsequent stimulation with cytokines (IL-12/IL-18) or combination stimulation (5-OP-RU + IL-12/IL-18) for 20 hours.

**Fig 9:**
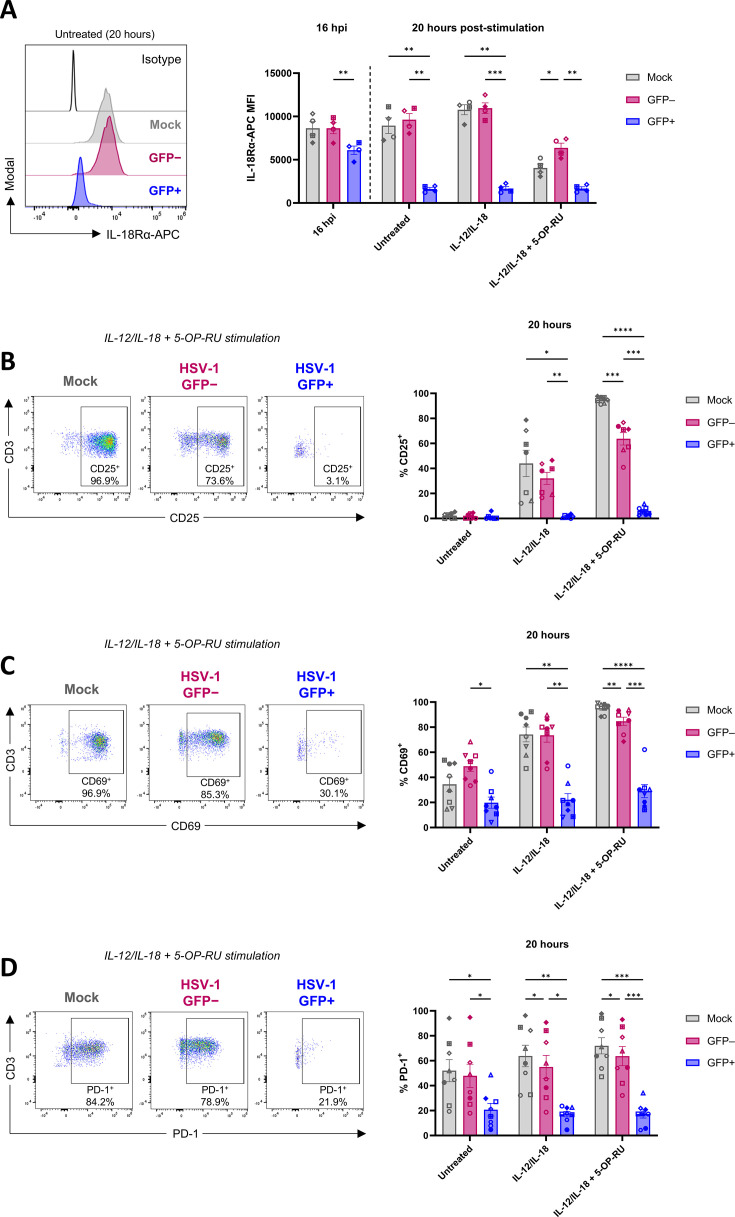
HSV-1 impairs IL-18Rα expression on MAIT cells and suppresses MAIT cell activation. HFF-hTERTs were mock-infected or infected with HSV-1 pICP47_GFP (MOI of 10) for 5 hours. Human PBMCs were co-cultured with mock- or HSV-1-infected fibroblasts for 16 hours. (**A**) PBMCs were harvested from co-culture after 16 hours (16 hpi) and were either stained for flow cytometry to assess IL-18Rα surface expression on MAIT cells (5-OP-RU-MR1 tetramer^+^CD3^+^ lymphocytes), or treated with either IL-12/IL-18 (both 50 ng/mL), or IL-12/IL-18 (both 50 ng/mL) + 5-OP-RU (10 nM) for 20 hours, prior to flow cytometry analysis of IL-18Rα surface expression on MAIT cells. MAIT cells from HSV-1-infected co-cultures were divided into GFP^+^ and GFP^–^ subsets for flow cytometry analysis. Representative histograms (left) display IL-18Rα cell-surface expression in mock (gray), HSV-1 GFP^–^ (pink), and HSV-1 GFP^+^ (blue) MAIT cells in the untreated control. The graph (right) displays IL-18Rα MFI in mock (gray), HSV-1 GFP^–^ (pink), and HSV-1 GFP^+^ (blue) MAIT cells (*n* = 4) for the indicated conditions. (**B–D**) Representative flow cytometry plots of IL-12/IL-18 + 5-OP-RU treatment (left) and graphs (right) display the percentage of mock (gray), HSV-1 GFP^–^ (pink), or HSV-1 GFP^+^ (blue) MAIT cells expressing (**B**) CD25 (*n* = 7), (**C**) CD69 (*n* = 8), and (**D**) PD-1 (*n* = 8), following the indicated stimulations. Symbols represent individual donors, and bars display mean ± SEM. Statistical significance was determined by repeated measures two-way ANOVA with Tukey’s multiple comparisons test comparing mock, GFP^–^, and GFP^+^ MAIT cells within each treatment group, *P < 0.05, **P < 0.01, ***P < 0.001, and ****P < 0.0001.

IL-18Rα is highly expressed on MAIT cells (36) (Fig. 9A). Across all conditions, including at 16 hpi (prior to stimulation), the IL-18Rα median fluorescence intensity (MFI) was significantly lower in GFP^+^ MAIT cells compared to GFP^–^ MAIT cells (Fig. 9A), with GFP^+^ MAIT cells displaying markedly lower IL-18Rα expression at the 20 hours post-stimulation timepoint. Compared to mock-infected MAIT cells, IL-18Rα expression was significantly lower in GFP^+^ MAIT cells in the 20-hour untreated (*P* = 0.0048) and IL-12/IL-18 treated condition (*P* = 0.0019; Fig. 9A). IL-18Rα expression levels were largely similar on GFP^–^ MAIT cells and mock-infected MAIT cells, with both populations displaying decreased IL-18Rα expression following IL-12/IL-18 + 5-OP-RU stimulation (2.2- and 1.5-fold decrease, respectively, relative to untreated). Expression of IL-18Rα was slightly higher on GFP^–^ MAIT cells relative to mock (*P* = 0.0414) and GFP^+^ MAIT cells (*P* = 0.0062) following IL-12/IL-18 + 5-OP-RU stimulation (Fig. 9A).

We also assessed the expression of IL-12 receptor β2 (IL-12Rβ2) subunit on mock-infected, GFP^–^, and GFP^+^ MAIT cells using flow cytometry. Although detection of IL-12β2 surface expression was relatively low across all conditions (Fig. S8), the MFI of IL-12Rβ2 was significantly higher in mock-infected relative to GFP^+^ MAIT cells after IL-12/IL-18 treatment (*P* = 0.0458) and in GFP^–^ compared to GFP^+^ MAIT cells after IL-12/IL-18 + 5-OP-RU treatment (*P* = 0.0327; Fig. S8). Together, these findings demonstrate that expression of the cytokine receptors IL-18R and IL-12R is suppressed on GFP^+^ MAIT cells.

Further analysis of MAIT cell surface phenotype following IL-12/IL-18 and IL-12/IL-18 + 5-OP-RU stimulation revealed the expression of CD25 (IL-2Rα), a component of the high-affinity IL-2 receptor complex, was markedly inhibited in GFP^+^ MAIT cells compared to both mock-infected and GFP^–^ MAIT cells in response to IL-12/IL-18 and combination stimulation (Fig. 9B). The percentage of MAIT cells expressing CD25 was also significantly lower in GFP^–^ MAIT cells (mean 63.7%, range 40.9%–76.7%) compared to mock-infected MAIT cells (mean 94.8%, range 91.2%–97.0%) following IL-12/IL-18 + 5-OP-RU treatment (*P* = 0.0007; Fig. 9B). The expression of other activation markers CD69 (Fig. 9C) and PD-1 (Fig. 9D) was also suppressed in GFP^+^ MAIT cells both at 16 hpi (prior to stimulation) and in response to IL-12/IL-18 and combination stimulation (Fig. 9C and D). Collectively, these results demonstrate that HSV-1 infection impaired MAIT cell activation and cytokine receptor expression, corresponding to reduced functional responses to stimulation.

## DISCUSSION

Here, we report that human peripheral blood MAIT cells are susceptible to direct infection by HSV-1 and show that HSV-1 profoundly impairs the ability of MAIT cells to functionally respond to both TCR-dependent and -independent signals. HSV-1-infected MAIT cells exhibit diminished pro-inflammatory cytokine and cytotoxic responses to activating stimuli, as well as decreased expression of key cytokine receptors necessary for TCR-independent and synergistic MAIT cell activation. Furthermore, the functional impacts of HSV-1 on MAIT cells extend beyond those that are directly infected, as MAIT cells that were exposed to HSV-1-infected fibroblasts in culture (but remained viral GFP negative) also exhibited a suppressed effector phenotype. Overall, these findings identify an immunomodulatory capability of HSV-1 to profoundly interfere with MAIT cell effector functions, by both directly infecting and indirectly affecting MAIT cells.

HSV-1 is a highly adapted human pathogen that employs multiple immunoevasive strategies to facilitate viral persistence and spread. Infection and functional inactivation of T cells by HSV-1 have been reported (14, 16, 70–72); however, these studies did not delineate conventional T cells from non-classical T cell populations, such as MAIT cells. MAIT cells are MR1-restricted innate-like lymphocytes that express a semi-invariant TRAV1-2^+^ TCR and are enriched in peripheral blood and mucosal tissues (23, 25). They can be rapidly activated by recognition of microbial riboflavin TCR ligands or innate cytokine signals, leading to the induction of effector functions such as cytotoxicity and cytokine production (24, 36, 73). As HSV-1 infects and replicates in mucocutaneous tissues, it is highly likely to encounter MAIT cells during initial infection or episodes of reactivation. The use of a 5-OP-RU-MR1 tetramer in this study allowed for the specific detection and analysis of MAIT cells following co-culture with HSV-1-infected cells *in vitro*. We identified that HSV-1 can infect multiple MAIT cell subsets and express IE, early, and late viral genes in infected MAIT cells. Furthermore, HSV-1-infected MAIT cells can transmit HSV-1 infection to fibroblasts. The finding that MAIT cells can be infected by HSV-1 adds to the growing understanding of interactions between MAIT cells and viruses (46, 47). The mechanism by which HSV-1 enters MAIT cells requires more investigation but may involve the transfer of HSV-1 through a virological synapse between infected fibroblasts and MAIT cells, as was demonstrated to occur in HSV-1 infection of conventional T cells (14). Verifying the expression and involvement of HSV-1 entry receptors, such as herpesvirus entry mediator (HVEM) and nectin-1 (14), on MAIT cells will also be valuable in understanding the basis of HSV-1 infection of MAIT cells.

Our results demonstrate that co-culture with HSV-1-infected fibroblasts led to substantial functional inhibition of infected MAIT cells, evidenced by the suppression of IFN-γ and TNF expression, and inhibition of degranulation and cytotoxic molecule expression (perforin and granzyme B). HSV-1 infection impaired MAIT cell effector responses to multiple modes of stimulation, including TCR-mediated activation with the potent riboflavin-related ligand 5-OP-RU, cytokine-mediated activation by IL-12/IL-18, and synergistic MAIT cell activation through the TCR and cytokines. Activation of MAIT cells via the TCR or cytokine signals elicits different transcriptional and functional profiles (35, 38) and allows MAIT cells to rapidly respond to different environmental cues that may be present during infections or inflammation. While it is currently unknown whether MAIT cells play a protective role in HSV-1 infection, there is increasing evidence of MAIT cell involvement in antiviral responses or immunopathology in a range of viral infections (30, 41, 44, 45). In addition to their direct effector functions such as cytotoxicity (43), MAIT cells can shape the responses of other immune subsets, such as by providing B cell help and promoting dendritic cell maturation (74–77). Thus, inhibition of multiple MAIT cell effector functions by HSV-1 could have significant consequences for the control of viral and other microbial infections, as MAIT cells impaired by HSV-1 infection are unable to secrete pro-inflammatory cytokines, upregulate cytotoxic molecules, or degranulate, in response to both TCR and cytokine signals.

Despite there being no currently known MR1 ligands synthesized by viruses (78), both cytokine and TCR modes of MAIT cell activation are relevant to consider in HSV-1 infection. HSV-1 infection predominantly manifests as lesions in the skin and mucosa, meaning that MAIT cell-activating antigens from resident riboflavin-synthesizing bacteria are likely a common presence at sites of HSV-1 infection. The suppression of MAIT cell functions by HSV-1 may provide an advantage to the virus by limiting immune cell activation in the local microenvironment. As MAIT cells can be activated by viral infections through secreted cytokines such as IL-12 and IL-18 (41), the diminished responsiveness of HSV-1-infected MAIT cells to IL-12/IL-18 and suppression of cytokine receptors such as IL-18R on MAIT cells may impact their ability to respond effectively to viral infections. The inhibition of IFN-γ and TNF production by MAIT cells could also conceivably aid the virus in establishing infection, as these cytokines are important in controlling HSV-1 infection (79, 80). Furthermore, HSV-1 has been associated with co-infections with other viruses, bacteria, and fungi, including riboflavin-synthesizing fungi such as *Candida albicans* (81–84). In a co-infection setting, the suppression of MAIT cell functions by HSV-1 may mean that MAIT cells are unable to aid in early antimicrobial immune responses to secondary pathogens. Thus, functional inhibition of MAIT cells by HSV-1 may have important implications for antiviral immunity and broader host defense.

Importantly, using an HSV-1 virus that expressed GFP under the control of an IE viral gene promoter for ICP47, which is among the first viral genes to be expressed following infection, we found that both HSV-1-infected (GFP^+^) MAIT cells as well as MAIT cells that were exposed to the virus in culture but did not indicate infection (GFP^–^) exhibited a suppressed effector response phenotype. It was recently reported that both *in vitro* VZV-infected (viral antigen positive) and VZV-exposed (viral antigen negative) MAIT cells also exhibit impaired functional responses to stimulation (48), identifying a shared ability of these related herpesviruses to impact MAIT cell responses. Interestingly, HSV-1-infected (GFP^+^) and HSV-1-exposed (GFP^–^) MAIT cells appear to be altered by HSV-1 in distinct modes. While both populations displayed impaired responses to stimulation with the TCR ligand 5-OP-RU, HSV-1-exposed MAIT cells were able to respond more effectively to IL-12/IL-18 stimulation than HSV-1-infected MAIT cells. In particular, IFN-γ production was profoundly inhibited in HSV-1 GFP^+^ MAIT cells in response to both TCR-dependent stimulation and IL-12/IL-18 stimulation, while HSV-1 GFP^–^ MAIT cells retained the ability to produce IFN-γ in response to IL-12/IL-18 stimulation, and unlike GFP^+^ MAIT cells, did not display reduced surface IL-18R expression. Entry of HSV-1 into conventional T cells has been shown to profoundly interfere with TCR signaling, resulting in reduced cytotoxic function, inhibiting proinflammatory cytokine production, and allowing selective synthesis of IL-10 (15, 17). In conventional T cells, the HSV-1 protein kinase and virion protein Us3 can disrupt TCR transduction by interfering with linker for activation of T cells (LAT) phosphorylation (15, 85). Exposure of iNKT cells to HSV-1-infected fibroblasts also resulted in impaired TCR signal transduction, with a decrease in phosphorylated extracellular signal-regulated kinase (ERK) (18). Future studies should evaluate the integrity of TCR signaling in both GFP^+^ and GFP^–^ MAIT cells following HSV-1 co-culture by investigating the phosphorylation of TCR signaling pathway proteins to determine if HSV-1-mediated interference with TCR signaling in MAIT cells may underlie the impaired responses to 5-OP-RU stimulation observed in the current study.

The suppression of MAIT cell effector functions did not appear to be mediated by a soluble factor in the supernatant of HSV-1-infected fibroblasts: PBMC co-cultures, as treatment of MAIT cells with supernatant from HSV-1-infected co-cultures did not result in functional suppression of MAIT cells, suggesting direct cell-to-cell contact with HSV-1-infected fibroblasts was required. Similarly, previous studies demonstrated that HSV-1-mediated functional impairment of conventional T cells and iNKT cells was dependent on direct contact with HSV-1-infected fibroblasts and did not require productive infection of T cells or iNKT cells (16, 18). Modulation of TCR signaling in conventional T cells was dependent upon viral entry but not viral gene expression or replication (15, 16). Thus, it is possible that HSV-1 entry into MAIT cells alone, without subsequent viral gene expression, may be capable of interfering with the functional capacity of MAIT cells. Interestingly, our results show that HSV-1-infected (GFP^+^) MAIT cells were inhibited to a greater degree than HSV-1-exposed (GFP^–^) MAIT cells across multiple effector outputs, suggesting direct infection with viral gene expression in MAIT cells is associated with more profound functional inhibition. The capacity of HSV-1 to modulate both infected (GFP^+^) and virally exposed (GFP^–^) cell populations has also been observed in studies of mature dendritic cells (mDCs), where reduced expression of CD83 and the IL-6 receptor was seen in both HSV-1 GFP^+^ and GFP^–^ mDCs (86, 87). This modulatory effect in GFP^–^ mDCs is thought to be partly mediated by HSV-1-derived non-infectious light particles generated during the infection of mDCs (86, 87). We also previously found that both HSV-1-infected (GFP^+^) and -exposed (GFP^–^) NK cells have an impaired ability to degranulate against target cells (49). The upregulation of inhibitory ligands on HSV-1-infected cells, or contact-dependent transfer of viral proteins, non-infectious viral particles, or extracellular vesicles, which deliver viral factors to uninfected cells (88), may represent other possible mechanisms by which HSV-1 might exert its immunomodulatory effects on virally exposed MAIT cells.

An important future avenue of research will be the comprehensive phenotypic and functional analysis of MAIT cells sampled from HSV-1 lesions during active disease to gain insight into whether MAIT cells are infected by HSV-1 *in vivo* and any consequential functional impairments. This may also provide valuable insight into how complex physiological settings, such as HSV-1 co-infection with MAIT cell activating bacteria, influence MAIT cell responses. Future studies could also explore whether HSV-1 impacts additional MAIT cell functions such as their tissue repair program (35), which may have relevance for wound healing associated with viral lesions.

In conclusion, we find that HSV-1 is capable of infecting human peripheral blood MAIT cells and inhibiting MAIT cell effector responses to TCR-dependent and cytokine-mediated stimulation. HSV-1 infection suppresses MAIT cell cytokine expression, cytotoxic potential, and cytokine receptor expression, with functional impairment in both virus-infected and virus-exposed MAIT cells. Collectively, these findings identify an additional immunomodulatory capacity of HSV-1 and, together with previous findings from our laboratory on HSV-1 modulation of MR1 (33, 34), underscore the need for further investigation of the significance and role of MAIT cells in HSV-1 infection. With recent interest in MAIT cells as targets for vaccine design (89–91), understanding how MAIT cells are modulated by ubiquitous viruses, such as HSV-1, may also bring important clues, and have important implications, for vaccine development.

## MATERIALS AND METHODS

### Cells and viruses

Human PBMCs from healthy adult donors were isolated by density gradient centrifugation with Ficoll-Paque PLUS (GE Healthcare) from buffy coats obtained from the Australian Red Cross Lifeblood under ethical approval (University of Sydney). PBMCs were cryopreserved in fetal calf serum (FCS; Serana) with 10% dimethylsulfoxide (Sigma-Aldrich) and were resuscitated and rested overnight in RPMI 1640 medium with L-glutamine (Thermo Fisher Scientific) supplemented with 10% human serum (Sigma-Aldrich) and 100 units/mL penicillin/streptomycin (Thermo Fisher Scientific) at 37°C with 5% CO_2_ prior to experiments.

HFF-hTERTs (92, 93) were maintained in Dulbecco’s modified Eagle medium (DMEM) with 4.5 g/L glucose and L-glutamine (Thermo Fisher Scientific), supplemented with 10% FCS and 100 units/mL penicillin/streptomycin (Thermo Fisher Scientific).

The following HSV-1 KOS strain recombinant viruses expressing enhanced green fluorescent protein (eGFP) were utilized: HSV-1 pICP47_eGFP/Cre (HSV-1 pICP47_GFP), HSV-1 pICP6_eGFP/Cre (HSV-1 pICP6_GFP), and HSV-1 pgB_eGFP/Cre (HSV-1 pgB_GFP) (50). HSV-1 strain KOS (courtesy Prof Paul Kinchington, University of Pittsburgh) was used in experiments assessing anti-HSV-1 gC antibody staining. Virus stocks were prepared and titrated on Vero cells.

### Infection of PBMCs with HSV-1

HFF-hTERTs were seeded in six-well plates at 3.5 × 10^5^ cells/well in supplemented DMEM and incubated overnight at 37°C, 5% CO_2_. HFF-hTERTs were then infected or mock-infected with cell-free HSV-1 pICP47_GFP (MOI = 10) and incubated for 1 hour with gentle rocking at 37°C with 5% CO_2_. Cells were then washed in phosphate-buffered saline (PBS) and incubated in fresh supplemented DMEM for 5 hours. Infection of HFF-hTERTs was verified by inverted fluorescence microscopy assessing GFP expression. After 5 hours, medium was removed, and PBMCs were added to mock- or HSV-1-infected HFF-hTERTs at a ratio of 1:3-1:5 (HFF-hTERT to PBMC) in supplemented RPMI 1640 medium. Co-cultures were spinoculated at 150 × *g* for 15 min at 37°C, then incubated at 37°C, 5% CO_2_ for 16 hours prior to harvest.

For the experiments comparing infection of MAIT cells between HSV-1 pICP47_GFP, HSV-1 pICP6_GFP, and HSV-1 pgB_GFP viruses, HFF-hTERTs were infected at an MOI of 5 for 20 hours, after which PBMCs were added to mock or infected HFF-hTERTs and co-cultured for 1 day prior to harvesting for flow cytometry analysis.

### MAIT cell stimulation following co-culture

PBMCs were harvested 16 hours post co-culture with mock or HSV-1 pICP47_GFP infected HFF-hTERTs and transferred to a 96-well plate in fresh supplemented RPMI 1640 medium. PBMCs were then stimulated with (i) 5-OP-RU (10 nM) (94) for 6 hours, (ii) IL-18 (R&D Systems) and IL-12 (Miltenyi Biotech) together (each 50 ng/mL) for 20 hours, or (iii) combined stimulation with 5-OP-RU (10 nM) plus IL-18 and IL-12 (each 50 ng/mL) for 20 hours. Unstimulated (untreated) controls were treated with dimethyl sulfoxide (DMSO) (1:10,000 [vol/vol]). For assessment of degranulation, anti-CD107a-APC antibody (clone H4A3; BioLegend; 1:80) or isotype control antibody was added for the duration of stimulation. Brefeldin A (5 µg/mL) and monensin (2 µM; both BioLegend) were added for the final 4 hours of stimulation in experiments assessing degranulation and intracellular cytokine expression. Cells were subsequently harvested for flow cytometry staining.

### Treatment of PBMCs with culture supernatant

HFF-hTERT cells were infected with HSV-1 pICP47_GFP (MOI of 10) for 5 hours as above, after which the medium was replaced with fresh supplemented RPMI 1640 medium with the addition of PBMCs. After co-culture between PBMCs and mock- or HSV-1 pICP47_GFP-infected HFF-hTERTs for 16 hours, co-culture supernatant was collected and centrifuged at 685 × *g* for 5 min to remove cells, then frozen at −80°C. Culture supernatants were also collected from mock- and HSV-1 pICP47_GFP-infected HFF-hTERTs cultured for 16 hours without the addition of PBMCs. When required, the supernatant was defrosted and centrifuged at 10,000 × *g* for 5 min at 4°C to remove debris. For assessment of MAIT cell infection after incubation in culture supernatant alone, PBMCs were incubated in mock or HSV-1 culture supernatants for 16 hours and then collected for flow cytometry analysis to assess GFP expression in MAIT cells. For functional assays, co-culture supernatant was diluted 1:1 in supplemented RPMI 1640 medium and incubated at 37°C, 5% CO_2_ for 24 hours with autologous PBMCs resuscitated 1 day prior. After incubation, supernatant-treated PBMCs were washed and stimulated with 5-OP-RU (10 nM) and/or IL-12/IL-18 (each 50 ng/mL), as described above.

### Flow cytometry

Cells were collected and stained with LIVE/DEAD Fixable Aqua viability dye (Thermo Fisher Scientific) and Human TruStain FcX Fc Receptor Blocking Solution (BioLegend) for 20 min at room temperature (RT). Cells were then washed in FACS buffer (PBS supplemented with 1% FCS and 10 mM EDTA) and stained with fluorescently conjugated surface antibodies for 30 min at 4°C. Cells were then washed in FACS buffer and either were fixed in BD Cytofix for 30 min at RT prior to acquisition, or were fixed/permeabilized with BD Cytofix/Cytoperm solution (BD Biosciences) for 20 min at 4°C for subsequent intracellular staining. Cells for intracellular staining were then washed in BD Perm/Wash buffer and stained with fluorescently conjugated intracellular antibodies in Perm/Wash buffer for 45 min at 4°C. For assessment of intracellular CC3, cells were stained with anti-CC3-PE antibody for 1 hour at RT. Finally, cells were washed in Perm/Wash buffer and resuspended in FACS buffer for acquisition. Data were acquired on a five-laser Aurora spectral cytometer (Cytek) or five-laser LSR II cytometer (BD Biosciences). For Aurora data, spectral unmixing using single-stained reference controls was performed using SpectroFlo software (Cytek). Subsequent compensation, where required, and manual gating were performed in FlowJo Software version 10.8.2 (BD Biosciences).

PBMCs were gated on lymphocytes (FSC-A vs SSC-A), single cells (FSC-A vs FSC-H), and live cells (LIVE/DEAD Fixable Aqua viability dye negative; Fig. S1A). MAIT cells were defined as CD3^+^ 5-OP-RU-MR1 tetramer^+^ lymphocytes (Fig. 1A; Fig. S1A). Other PBMC lymphocyte populations were defined as follows: CD4^+^ T cells (CD3^+^ 5-OP-RU-MR1 tetramer^−^ CD56^−^ CD4^+^ CD8^−^), CD8^+^ T cells (CD3^+^ 5-OP-RU-MR1 tetramer^−^ CD56^−^ CD4^−^ CD8^+^), and non-MAIT CD3^+^CD56^+^ lymphocytes (CD3^+^ 5-OP-RU-MR1 tetramer^−^ CD56^+^; Fig. 1A). For the assessment of intracellular CC3 staining by flow cytometry, MAIT cells were identified as CD3^+^ TCR Vα7.2^+^ CD161^high^ lymphocytes (Fig. S4A) (19) as the anti-CC3 antibody and MR1 tetramer were conjugated to the same fluorophore (PE) so could not be used simultaneously in this assay.

### Flow cytometry antibodies

The following fluorophore-conjugated antibodies were used for flow cytometry surface staining: anti-CD3-BUV395 (UCHT1; BD Biosciences; 1:40 [vol/vol]), 5-OP-RU loaded MR1 tetramer-PE (A/Prof. Alexandra Corbett, The University of Melbourne; 1:500), anti-CD56-BV605 (NCAM16.2; BD Biosciences; 1:50), anti-CD4-BUV805 (SK3; BD Biosciences; 1:40), anti-CD8a-AlexaFluor700 (SK1; BioLegend; 1:80), anti-CD69-PerCP/Cy5.5 (FN50; BioLegend; 1:40), anti-PD-1-PE-Dazzle594 (EH12.2H7; BioLegend; 1:28), anti-IL-18Rα (CD218a)-APC (H44; BioLegend; 1:20), anti-IL-12Rβ2-BV421 (S16020B; BioLegend; 1:20), anti-CD25-BV786 (M-A251; BD Biosciences; 1:25), anti-CD161-PE/Dazzle594 (HP-3G10; Biolegend; 1:40), anti-TCR Vα7.2-PE/Cy7 (3C10; BioLegend; 1:40), and anti-HSV-1 gC-FITC (0143; Virostat; 1:20). The following fluorophore-conjugated antibodies were used for intracellular flow cytometry: anti-granzyme B-PE-Cy7 (QA18A28; BioLegend; 1:40), anti-perforin-APC/Fire750 (B-D48; BioLegend; 1:40), anti-IFN-γ-BV421 (4S.B3; BioLegend; 1:28), anti-TNF-BV785 (MAb11; BioLegend; 1:50), and anti-active caspase-3 (CC3)-PE (C92-605; BD Biosciences; 1:20). Isotype control antibodies were used as appropriate. BD Horizon Brilliant Stain Buffer Plus (BD Biosciences) was included in antibody mastermixes when two or more BD Brilliant dyes were used.

### Isolation of MAIT cells by fluorescence-activated cell sorting

To isolate MAIT cells from total PBMCs using FACS, human PBMCs were stained with LIVE/DEAD Fixable Aqua viability dye (Thermo Fisher Scientific) and Human TruStain FcX Fc Receptor Blocking Solution (BioLegend) for 20 min at RT. Cells were then stained with anti-CD3-BV421 (UCHT1; BD Biosciences; 1:28) and 5-OP-RU loaded MR1 tetramer-PE (1:500) for 30 min at 4°C. MAIT cells, identified as CD3^+^ 5-OP-RU-MR1 tetramer^+^ viability dye-negative lymphocytes, were then isolated from whole PBMCs by FACS on a seven-laser BD Influx cell sorter, to >95% purity. MAIT cells were collected in FCS and washed prior to experiments.

### Infection of purified MAIT cells

HFF-hTERTs were labeled with 1 µM CellTrace Violet (CTV; Invitrogen) and seeded in 48-well plates at 4.3 × 10^4^ cells/well in supplemented DMEM 1 day prior to infection with HSV-1 pICP47_GFP (MOI of 10). After viral adsorption for 1 hour at 37°C, 5% CO_2_, the medium was replaced with fresh supplemented DMEM, and HFF-hTERTs were incubated for a further 5 hours. MAIT cells were isolated from whole PBMCs by FACS and added to HSV-1-infected HFF-hTERTs at 1.5–2.5 × 10^5^ MAIT cells/well in supplemented RPMI 1640 medium. Co-cultures were spinoculated at 150 × *g* for 15 min at 37°C and then incubated for 16 hours at 37°C, 5% CO_2_. MAIT cells were collected for flow cytometry analysis to assess viral GFP expression, and HFF-hTERTs were excluded on the basis of CTV staining and forward/side scatter characteristics.

For visualization of GFP nuclear fluorescence localization, MAIT cells were purified from whole PBMCs by FACS and then infected with HSV-1 pICP47_GFP for 16 hours as above. MAIT cells were then collected, washed, and spotted onto microscope slides. Cell spots were left to dry at RT for 20 min, then were fixed with 4.2% formaldehyde (BD Cytofix Fixation Buffer; BD Biosciences) for 20 min at RT, washed twice in Dulbecco's phosphate-buffered saline (DPBS), and stained with DAPI ready-made solution (Sigma-Aldrich) for 20 min at RT. Coverslips were applied, and images were acquired using an Olympus BX51 fluorescence microscope with a DP73 camera (Olympus).

### Infectious center assay

HFF-hTERT (3.5 × 10^5^ cells/well) in six-well plates were infected with HSV-1 pICP47_GFP (MOI = 10) for 5 hours prior to the addition of human PBMCs (3 × 10^6^ cells/well). After 16 hours of co-culture, PBMCs were collected and stained, and GFP^+^ MAIT cells (CD3^+^ 5-OP-RU-MR1 tetramer^+^ live lymphocytes) were isolated by FACS to >95% purity using a seven-laser BD Influx cell sorter. GFP^+^ MAIT cells were then treated with low pH citrate buffer (40 mM C_6_H_5_O_7_Na_3_, 135 mM NaCl, and 10 mM KCl; pH 3) for 2 min at RT to inactivate unpenetrated virus, then washed twice in supplemented DMEM. GFP^+^ MAIT cells (3 × 10^3^), in triplicate per donor, were added to uninfected HFF-hTERT monolayers pre-seeded on sterile, circular glass coverslips (6.5 × 10^4^ HFF-hTERT/coverslip) in 24-well plates. After the addition of GFP^+^ MAIT cells and incubation in supplemented DMEM at 37°C, 5% CO_2_ for 1 day, medium was removed, and coverslips were gently washed in DPBS and fixed in BD Cytofix Fixation Buffer (contains 4.2% formaldehyde) for 20 min at RT. Coverslips were washed in DPBS before being mounted with ProLong Gold Antifade Mountant with DAPI (Invitrogen) on glass microscope slides. GFP^+^ infectious centers were imaged using an Olympus BX51 fluorescence microscope with an Olympus DP73 camera and cellSens Standard 1.12 (Olympus) software, with representative images captured from each donor. Pseudo-flat field correction was applied in ImageJ (version 1.54 j) to correct for uneven illumination in the DAPI channel.

### Statistical analysis

Statistical analyses were performed in GraphPad Prism version 10.1 (GraphPad Software, LLC). Statistical significance was assessed by repeated measures one- or two-way ANOVA with Tukey’s multiple comparisons test, repeated measures two-way ANOVA with Šídák’s multiple comparisons test, or two-tailed paired *t*-test, as indicated in figure legends. *P* < 0.05 was considered significant.

## Data Availability

The data underlying this study are available from the corresponding author upon reasonable request.

## SUPPLEMENTAL MATERIAL

Figure S1 mbio.03887-24-s0001.pdf Flow cytometry gating strategy and frequency of MAIT cells.

Figure S2 mbio.03887-24-s0002.pdf HSV-1 infects purified peripheral blood-derived MAIT cells.

Figure S3 mbio.03887-24-s0003.pdf Frequency of MAIT cell subpopulations.

Figure S4 mbio.03887-24-s0004.pdf Viability of CD56^+^ and CD56^−^ MAIT cells following HSV-1 infection.

Figure S5 mbio.03887-24-s0005.pdf Incubation of PBMCs with supernatant from HSV-1-infected cultures did not lead to detectable GFP expression in MAIT cells.

Figure S6 mbio.03887-24-s0006.pdf Nuclear localization of GFP fluorescence in MAIT cells infected with HSV-1 pICP47_GFP.

Figure S7 mbio.03887-24-s0007.pdf Comparison of HSV-1 glycoprotein C staining and viral promoter-driven GFP in peripheral blood T cell populations after co-culture with HSV-1-infected fibroblasts.

Figure S8 mbio.03887-24-s0008.pdf Surface expression of IL‐12Rβ2 in mock‐infected, HSV‐1 GFP^−^, and HSV‐1 GFP^+^ MAIT cells.

ASM does not own the copyrights to Supplemental Material that may be linked to, or accessed through, an article. The authors have granted ASM a non-exclusive, world-wide license to publish the Supplemental Material files. Please contact the corresponding author directly for reuse.
