# Supplementary material for: Herpes simplex virus type 1 impairs mucosal-associated invariant T cells
Source: mBio. 2025 Mar 26;16(5):e03887-24. doi: 10.1128/mbio.03887-24 (PMC12077205; doi:10.1128/mbio.03887-24)
Supplement: Figure S1 — Flow cytometry gating strategy and frequency of MAIT cells. [file mbio.03887-24-s0001.pdf]

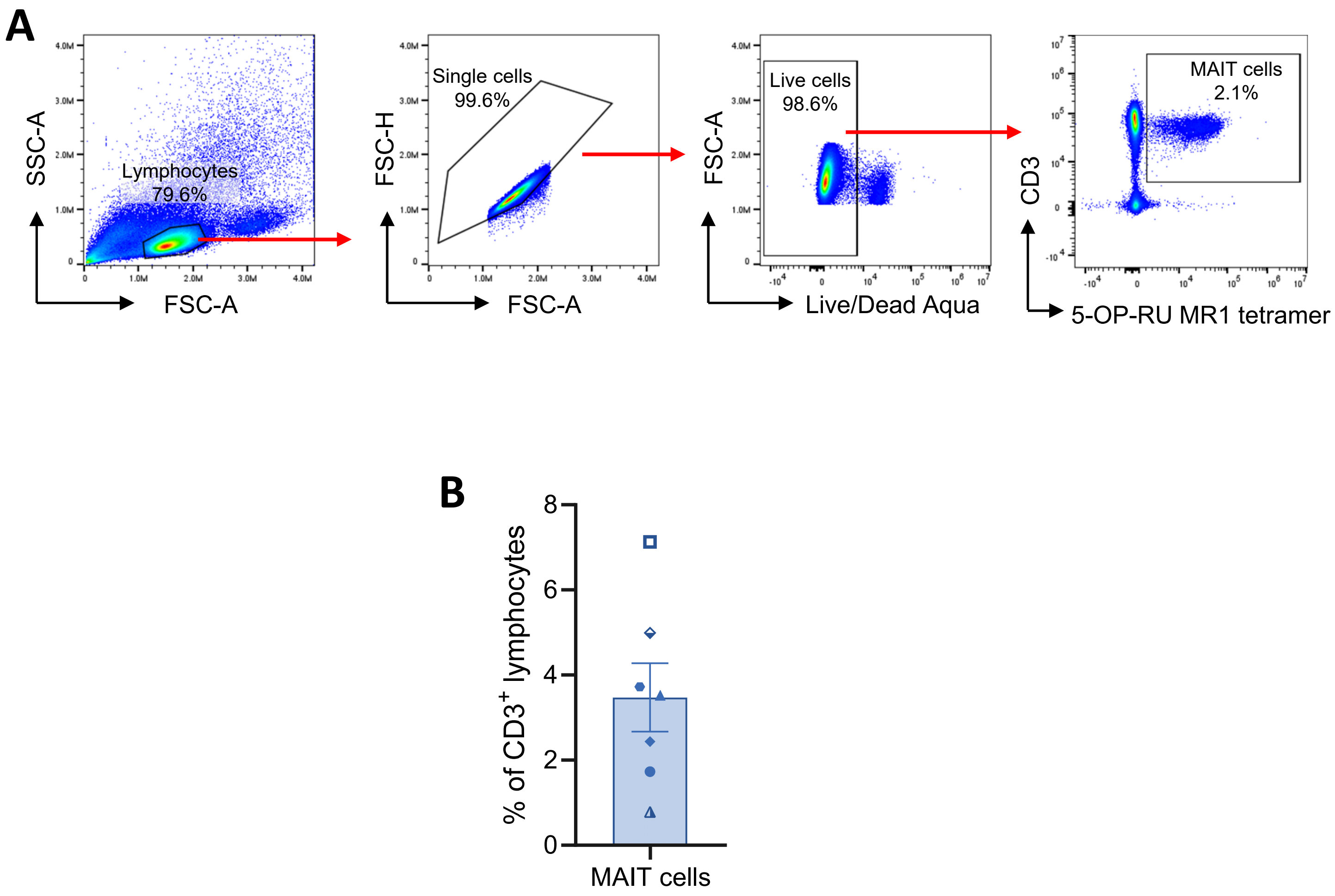

<u>Supplementary Figure 1.</u> Flow cytometry gating strategy and frequency of mucosal associated invariant T (MAIT) cells

(A) Representative flow cytometry gating strategy for human peripheral blood mononuclear cells (PBMCs), beginning with selection of lymphocytes, then single cells, then live cells (based on exclusion of LIVE/DEAD™ Fixable Aqua viability dye) prior to gating for specific lymphocyte subsets such as MAIT cells ($CD3^+$ 5-OP-RU MR1 $tetramer^+$). **(B)** MAIT cells as a percentage of live $CD3^+$ lymphocytes. Symbols represent individual donors (n=7), with mean ± SEM shown.
