# Supplementary material for: Herpes simplex virus type 1 impairs mucosal-associated invariant T cells
Source: mBio. 2025 Mar 26;16(5):e03887-24. doi: 10.1128/mbio.03887-24 (PMC12077205; doi:10.1128/mbio.03887-24)
Supplement: Figure S2 — HSV-1 infects purified peripheral blood-derived MAIT cells. [file mbio.03887-24-s0002.pdf]

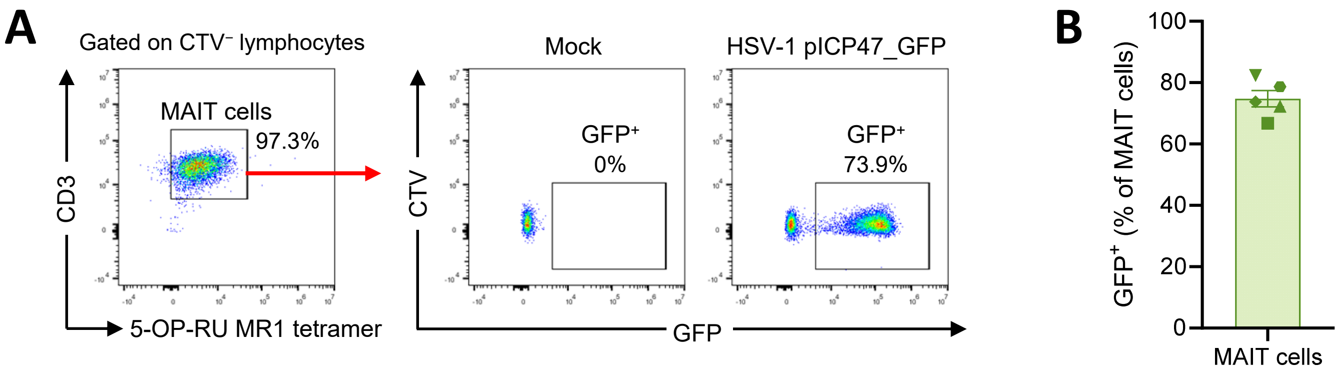

<u>Supplementary Figure 2.</u> HSV-1 infects purified peripheral blood-derived MAIT cells

Mucosal associated invariant T (MAIT) cells ($CD3^+$ 5-OP-RU-MR1 $tetramer^+$ viability dye-negative lymphocytes) were isolated from whole human peripheral blood mononuclear cell (PBMCs) samples by fluorescence-activated cell sorting and co-cultured for 16 hours with CellTrace Violet (CTV)-labelled mock-infected or HSV-1 pICP47_GFP-infected human telomerase reverse transcriptase immortalised human foreskin fibroblasts (HFF-hTERT) at a ratio of 1 HFF-hTERT : 3-5 MAIT cells. HFF-hTERTs were infected with HSV-1 pICP47_GFP for 5 hours (MOI of 10) prior to the addition of purified MAIT cells. After 16 hours, MAIT cells were harvested from co-culture and the percentage of MAIT cells (5-OP-RU-MR1 $tetramer^+CD3^+$ CTV^- lymphocytes) expressing GFP was assessed by flow cytometry. **(A)** Representative flow cytometry gating strategy for GFP detection in mock-infected and HSV-1 pICP47_GFP-infected MAIT cells. **(B)** The percentage of MAIT cells expressing GFP after 16 hours of co-culture with HSV-1 pICP47_GFP infected HFF-hTERTs. Symbols represent individual donors (n=5), mean ± SEM shown.
