# Supplementary material for: Herpes simplex virus type 1 impairs mucosal-associated invariant T cells
Source: mBio. 2025 Mar 26;16(5):e03887-24. doi: 10.1128/mbio.03887-24 (PMC12077205; doi:10.1128/mbio.03887-24)
Supplement: Figure S3 — Frequency of MAIT cell subpopulations. [file mbio.03887-24-s0003.pdf]

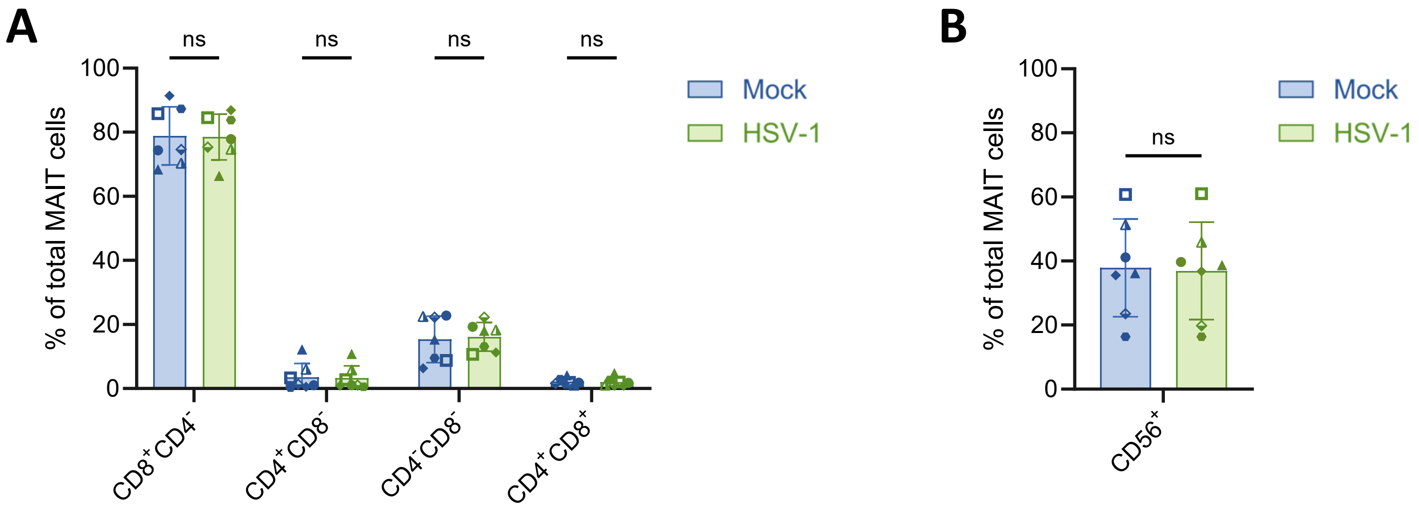

<u>Supplementary Figure 3.</u> Frequency of mucosal associated invariant T (MAIT) cell subpopulations
Frequency of **(A)** $CD8^+CD4^–$, $CD4^+CD8^–$, $CD8^–CD4^–$ and $CD8^+CD4^+$ MAIT cell subsets, and **(B)** $CD56^+$ MAIT cells, expressed as a percentage of total MAIT cells (5-OP-RU-MR1 $tetramer^+CD3^+$ lymphocytes), following 16 hours of co-culture with mock-infected (blue) or HSV-1 pICP47_GFP-infected (green) human telomerase reverse transcriptase immortalised human foreskin fibroblasts (HFF-hTERTs). Symbols represent individual donors (n=7). Bars display mean ± SEM. Statistical significance evaluated by **(A)** repeated measures two-way ANOVA with Šídák’s multiple comparisons test comparing mock and HSV-1 per subset, or **(B)** two-tailed paired *t*-test comparing mock and HSV-1. ns; non-significant (P>0.05).
