# Supplementary material for: Herpes simplex virus type 1 impairs mucosal-associated invariant T cells
Source: mBio. 2025 Mar 26;16(5):e03887-24. doi: 10.1128/mbio.03887-24 (PMC12077205; doi:10.1128/mbio.03887-24)
Supplement: Figure S4 — Viability of CD56+ and CD56− MAIT cells following HSV-1 infection. [file mbio.03887-24-s0004.pdf]

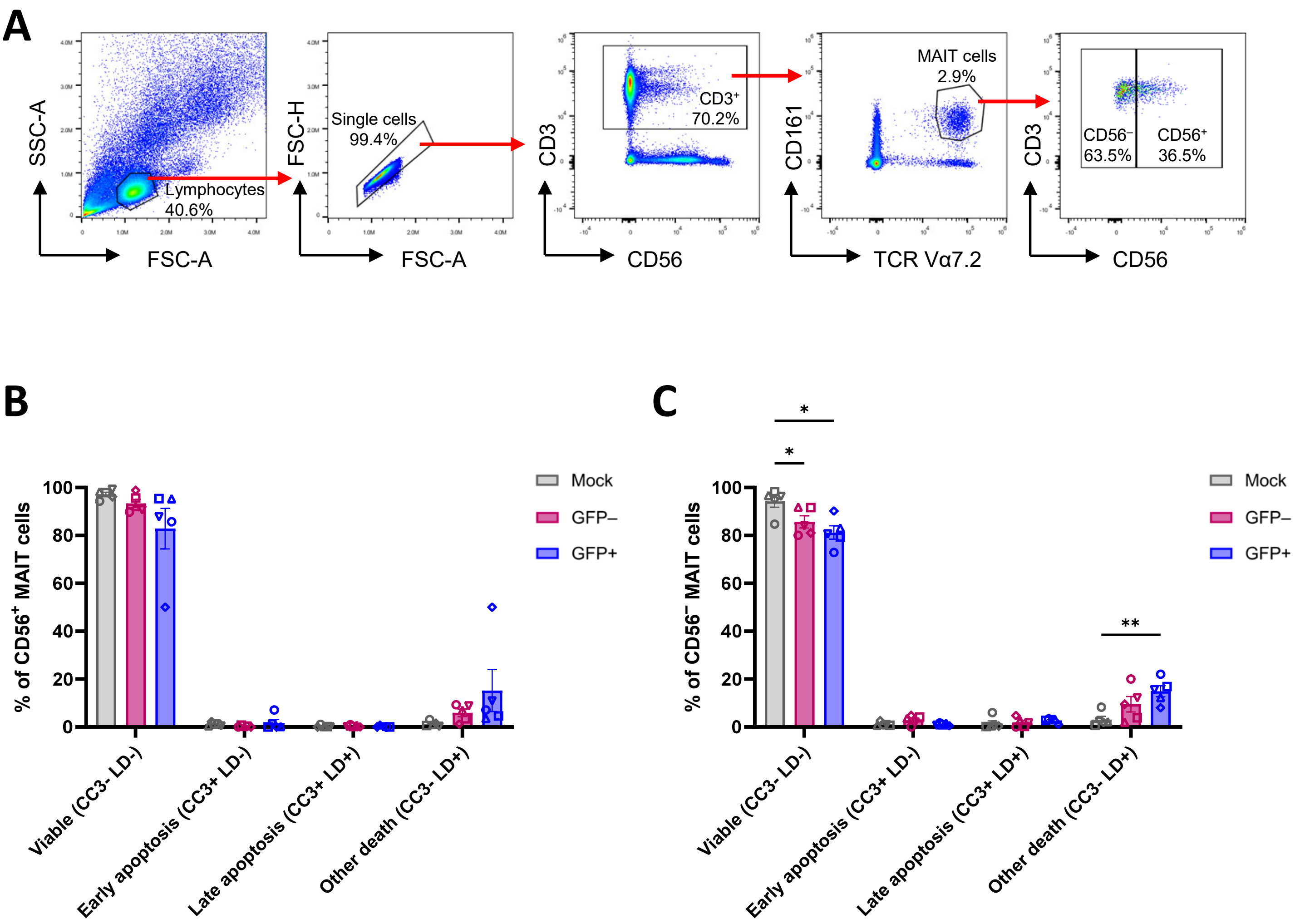

Supplementary Figure 4. Viability of CD56⁺ and CD56⁻ MAIT cells following HSV-1 infection

Human peripheral blood mononuclear cells (PBMCs) were co-cultured with mock-infected or HSV-1 pICP47_GFP-infected HFF-hTERT cells for 16 hours. PBMCs were then harvested and stained with Live/Dead Aqua (LD) viability dye and intracellular anti-cleaved caspase 3 (CC3) antibody for assessment of cell death via flow cytometry. **(A)** Representative gating strategy for identification of mucosal-associated invariant T (MAIT) cells in this assay. MAIT cells were identified as $CD3^+$ TCR Vα7.2$^+$ $CD161^{high}$ lymphocytes and were divided into $CD56^+$ and $CD56^-$ subsets by flow cytometry gating. **(B-C)** The percentage of viable cells (LD^- $CC3^-$), early apoptotic cells (LD^- $CC3^+$), late apoptotic cells (LD^+ $CC3^+$), and non-apoptotic non-viable cells (‘other’ death; LD^+ $CC3^-$) was measured in mock-infected (grey), HSV-1 GFP^+ (blue) and HSV-1 GFP^- (pink) cells in **(B)** $CD56^+$ and **(C)** $CD56^-$ MAIT cell subpopulations. Symbols represent individual donors (n=5). Bars show mean ± SEM. Statistical significance determined by repeated measures two-way ANOVA with Tukey’s multiple comparisons test, *P<0.05, **P<0.01.
