# Supplementary material for: Herpes simplex virus type 1 impairs mucosal-associated invariant T cells
Source: mBio. 2025 Mar 26;16(5):e03887-24. doi: 10.1128/mbio.03887-24 (PMC12077205; doi:10.1128/mbio.03887-24)
Supplement: Figure S5 — Incubation of PBMCs with supernatant from HSV-1-infected cultures did not lead to detectable GFP expression in MAIT cells. [file mbio.03887-24-s0005.pdf]

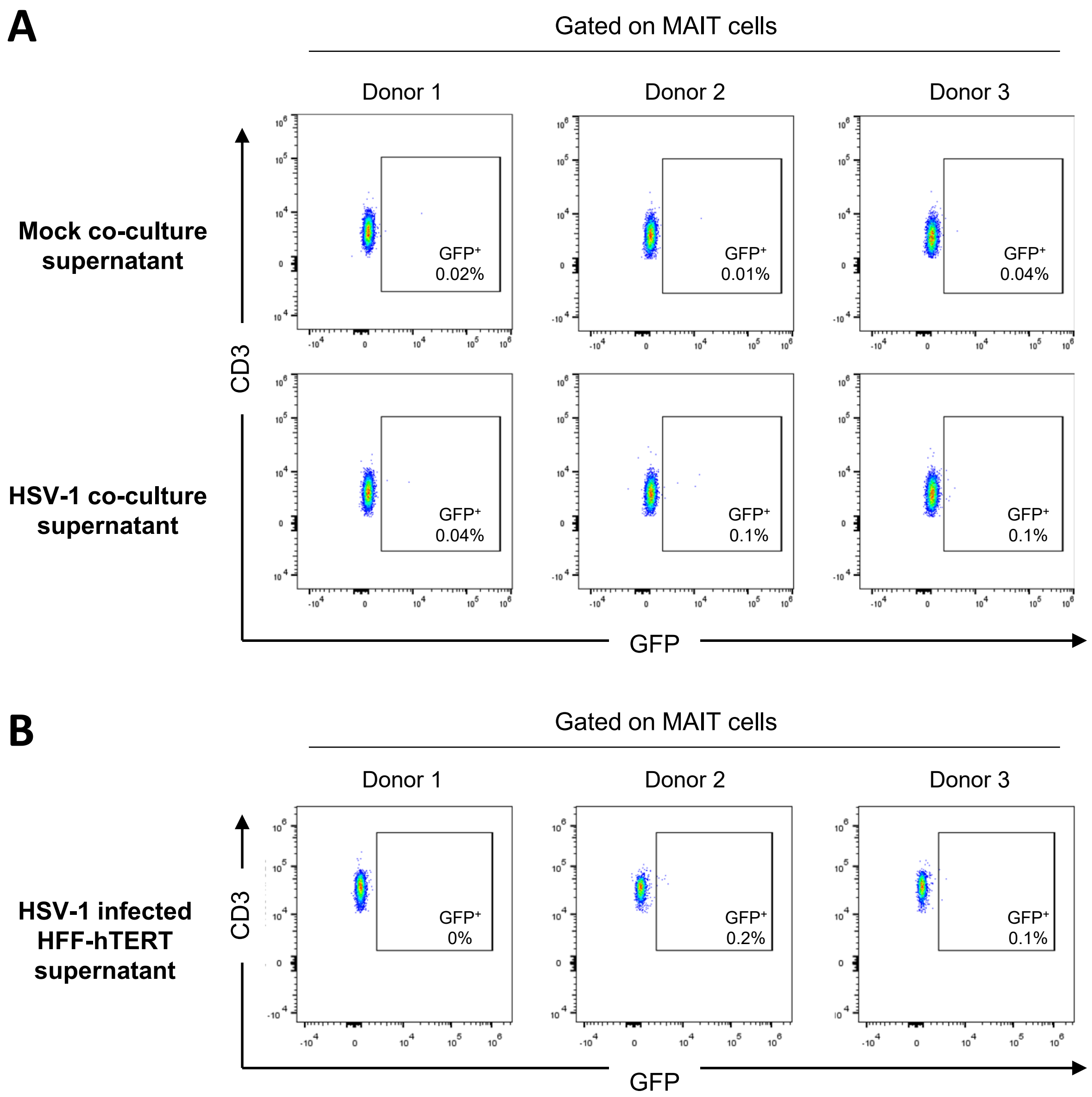

<u>Supplementary Figure 5.</u> Incubation of PBMCs with supernatant from HSV-1-infected cultures did not lead to detectable GFP expression in MAIT cells

Culture supernatant was collected from mock- or HSV-1 pICP47_GFP-infected human telomerase reverse transcriptase immortalised human foreskin fibroblasts (HFF-hTERTs) cultured with or without human peripheral blood mononuclear cells (PBMCs) for 16 hours. **(A)** Fresh PBMCs were incubated for one day with supernatant collected from mock- or HSV-1-infected fibroblast:PBMC co-cultures. PBMCs were then stimulated with IL-12/IL-18 and/or 5-OP-RU and flow cytometry analysis performed to assess the percentage of MAIT cells (5-OP-RU-MR1 tetramer$^+$CD3$^+$ lymphocytes) expressing GFP. Representative flow cytometry plots from three PBMC donors (gated on MAIT cells) of IL-12/IL-18 treatment (50 ng/mL each) for 20 hours, displaying the percentage of GFP$^+$ MAIT cells. **(B)** Representative flow cytometry plots from three PBMC donors show the percentage of MAIT cells expressing GFP after incubating fresh PBMCs for 16 hours in culture supernatant collected from HSV-1-infected fibroblasts.
