# Supplementary material for: Herpes simplex virus type 1 impairs mucosal-associated invariant T cells
Source: mBio. 2025 Mar 26;16(5):e03887-24. doi: 10.1128/mbio.03887-24 (PMC12077205; doi:10.1128/mbio.03887-24)
Supplement: Figure S6 — Nuclear localization of GFP fluorescence in MAIT cells infected with HSV-1 pICP47_GFP. [file mbio.03887-24-s0006.pdf]

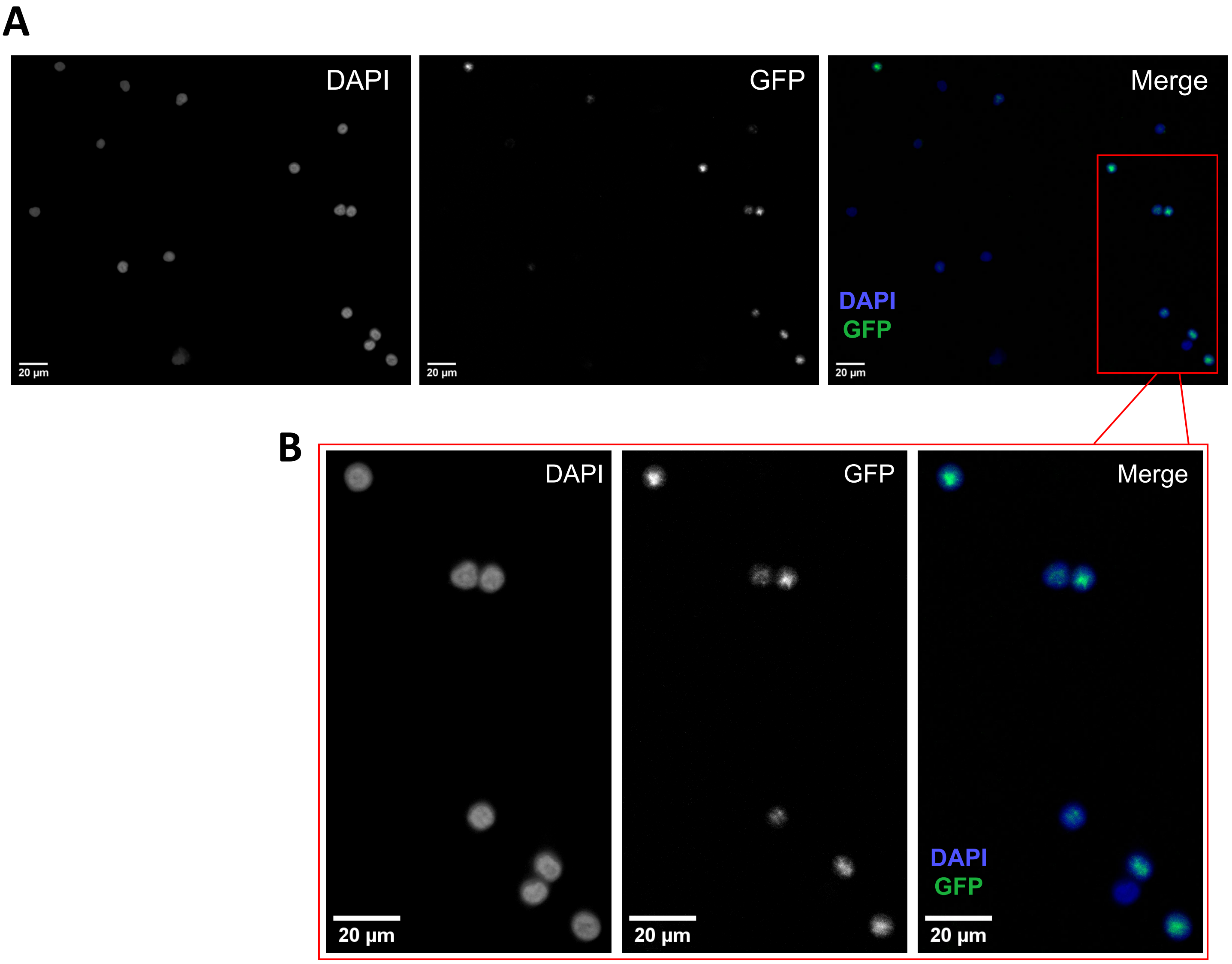

Supplementary Figure 6. Nuclear localisation of GFP fluorescence in MAIT cells infected with HSV-1 pICP47_GFP

Mucosal-associated invariant T (MAIT) cells (CD3$^+$ 5-OP-RU-MR1 tetramer$^+$ viability dye-negative lymphocytes) were isolated from human peripheral blood mononuclear cell (PBMCs) samples by fluorescence-activated cell sorting. Isolated MAIT cells were then co-cultured for 16 hours with HSV-1 pICP47_eGFP/Cre-infected HFF-hTERTs (infected at MOI of 10 for 5 hours prior to addition of MAIT cells) at a ratio of 1 HFF-hTERT : 3-5 MAIT cells. After 16 hours, MAIT cells were collected, spotted onto microscope slides, fixed and counterstained with DAPI. Fluorescence microscopy was used to visualise GFP and DAPI. **(A)** Representative cell spots of MAIT cells from one donor, showing single channel images for DAPI and GFP in grey, and merge image of DAPI (blue) and GFP (green). The region identified in the red box is shown enlarged in **(B)**, with merge image showing DAPI (blue) and GFP (green). Images are representative of three independent donors.
