# Supplementary material for: Herpes simplex virus type 1 impairs mucosal-associated invariant T cells
Source: mBio. 2025 Mar 26;16(5):e03887-24. doi: 10.1128/mbio.03887-24 (PMC12077205; doi:10.1128/mbio.03887-24)
Supplement: Figure S7 — Comparison of HSV-1 glycoprotein C staining and viral promoter-driven GFP in peripheral blood T cell populations after co-culture with HSV-1-infected fibroblasts. [file mbio.03887-24-s0007.pdf]

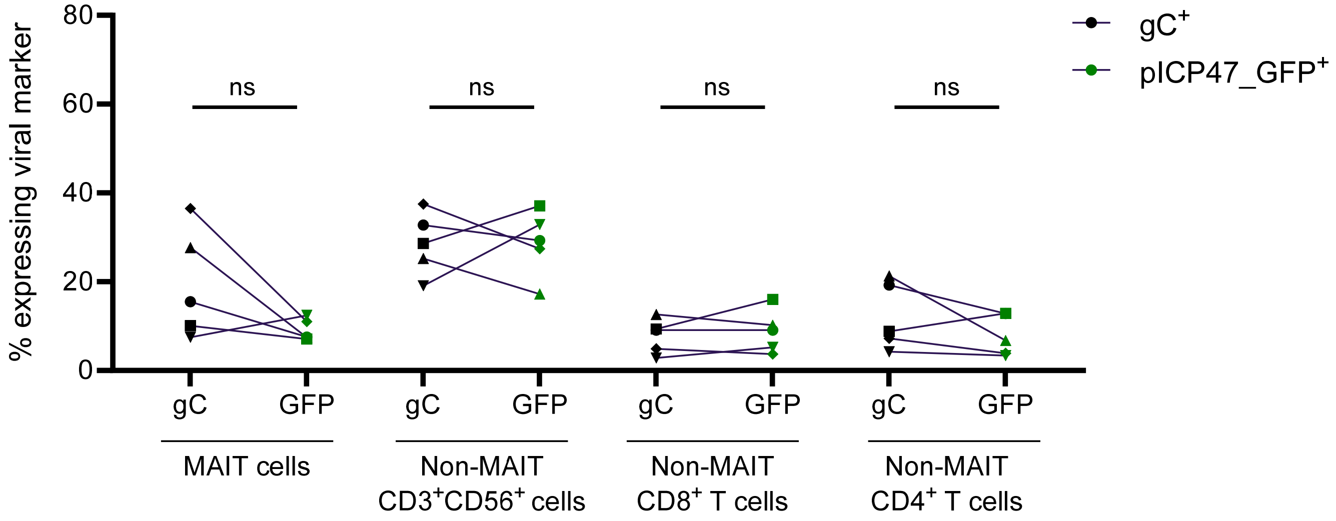

Supplementary Figure 7. Comparison of HSV-1 glycoprotein C staining and viral promoter-driven GFP in peripheral blood T cell populations after co-culture with HSV-1-infected fibroblasts

Human telomerase reverse transcriptase immortalised human foreskin fibroblasts (HFF-hTERT) were infected with either HSV-1 strain KOS or HSV-1 KOS pICP47_GFP (MOI of 10) for 5 hours. Human peripheral blood mononuclear cells (PBMCs) were then co-cultured with infected or mock-infected HFF-hTERTs for 16 hours and collected for flow cytometry analysis. PBMCs incubated with HSV-1 KOS-infected HFF-hTERTs were stained with a fluorescein isothiocyanate (FITC)-conjugated anti-HSV-1 glycoprotein C (gC) antibody as a marker of infection. For PBMCs incubated with HSV-1 pICP47_GFP-infected HFF-hTERTs, GFP was used as a marker of infection. Graph depicts the percentage of mucosal associated invariant T (MAIT) cells ($CD3^+$ 5-OP-RU-MR1 $tetramer^+$ lymphocytes), non-MAIT $CD3^+CD56^+$ cells (MR1 $tetramer^-CD3^+CD56^+$ lymphocytes), non-MAIT $CD8^+$ T cells (MR1 $tetramer^-CD3^+CD56^-CD4^-CD8^+$ lymphocytes) and non-MAIT $CD4^+$ T cells (MR1 $tetramer^-CD3^+CD56^-CD4^+CD8^-$ lymphocytes) expressing HSV-1 gC (black symbols) or GFP (green symbols) after 16 hours of viral co-culture. Symbols and lines indicate individual PBMC donors (n=5). Statistical significance evaluated by two-tailed paired *t*-test per subset. ns, not significant ($P>0.05$).
