# Supplementary material for: Herpes simplex virus type 1 impairs mucosal-associated invariant T cells
Source: mBio. 2025 Mar 26;16(5):e03887-24. doi: 10.1128/mbio.03887-24 (PMC12077205; doi:10.1128/mbio.03887-24)
Supplement: Figure S8 — Surface expression of IL‐12Rβ2 in mock‐infected, HSV‐1 GFP−, and HSV‐1 GFP+ MAIT cells. [file mbio.03887-24-s0008.pdf]

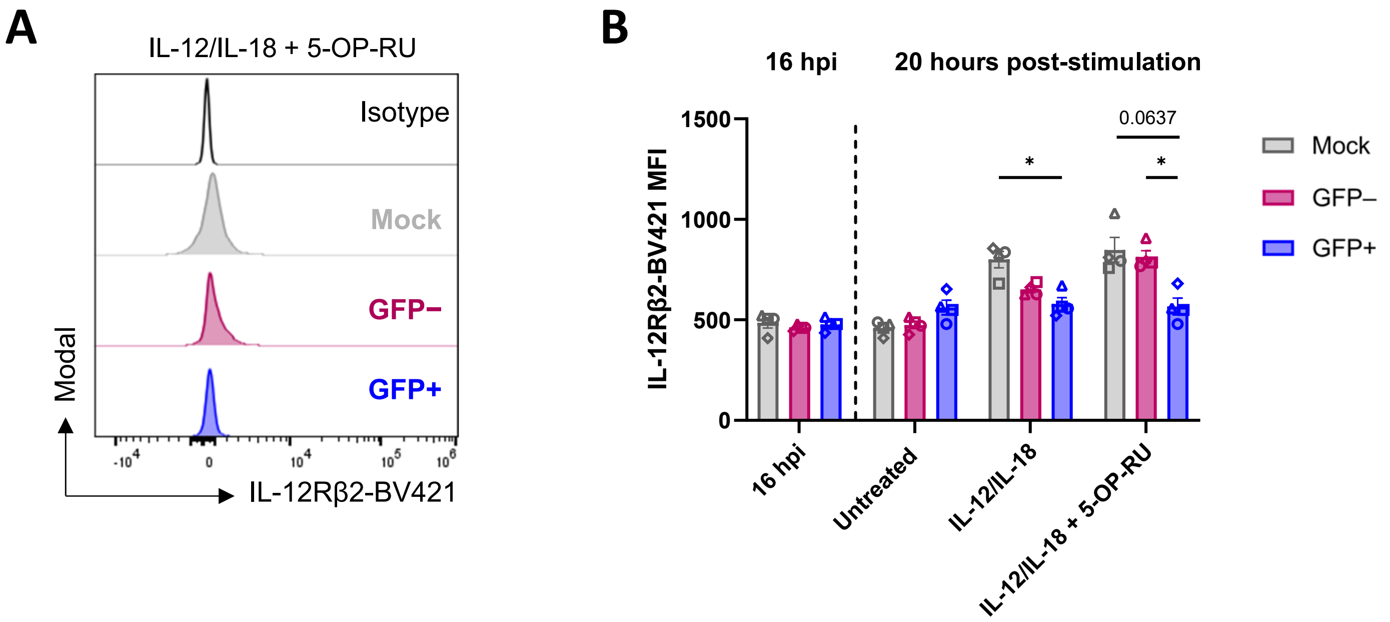

<u>Supplementary Figure 8</u>. Surface expression of IL-12Rβ2 in mock-infected, HSV-1 GFP⁻ and HSV-1 GFP⁺ mucosal associated invariant T (MAIT) cells

Human peripheral blood mononuclear cells (PBMCs) were co-cultured with mock- or HSV-1 pICP47_GFP-infected human telomerase reverse transcriptase immortalised human foreskin fibroblasts (HFF-hTERTs) for 16 hours. PBMCs were then harvested from co-culture after 16 hours (16 hours post-infection) and were either stained for flow cytometry to assess IL-12Rβ2 surface expression on MAIT cells, or treated with either IL-12/IL-18 (both 50 ng/ml), or IL-12/IL-18 (both 50 ng/ml) + 5-OP-RU (10 nM) for 20 hours, prior to flow cytometry analysis of IL-12Rβ2 surface expression on MAIT cells. MAIT cells ($CD3^{+}$ 5-OP-RU-MR1 $tetramer^{+}$ lymphocytes) from HSV-1-infected co-cultures were partitioned into GFP^{+} and GFP^{-} subsets for flow cytometry analysis. **(A)** Representative histograms display IL-12Rβ2 surface expression in mock (grey), HSV-1 GFP^{-} (pink), HSV-1 GFP^{+} (blue) MAIT cells from one donor following IL-12/IL-18 + 5-OP-RU treatment. **(B)** Graph shows IL-12Rβ2 median fluorescence intensity (MFI) in mock (grey), HSV-1 GFP^{-} (pink), HSV-1 GFP^{+} (blue) MAIT cells. Symbols represent individual donors (n=4), bars display mean ± SEM. Statistical significance determined by repeated measures two-way ANOVA with Tukey's multiple comparisons test, *$P<0.05$. hpi, hours post-infection.
